# Association Between Ambient Air Pollution and Attention-Deficit/Hyperactivity Disorder (ADHD) in Children: A Systematic Review and Meta-Analysis

**DOI:** 10.7759/cureus.71527

**Published:** 2024-10-15

**Authors:** Shamshad Ahmad, Naveen K G, Arun Mani Babu, Rajeev Ranjan, Pragya Kumar

**Affiliations:** 1 Community and Family Medicine, All India Institute of Medical Sciences, Patna, Patna, IND; 2 Psychiatry, All India Institute of Medical Sciences, Patna, Patna, IND

**Keywords:** air-contamination, ambient air pollution, attention-deficit/hyperactivity disorder (adhd), children adhd, pollutant concentrations

## Abstract

The objective of this systematic review and meta-analysis was to assess the association between postnatal exposure to ambient air pollutants (particulate matter (PM)_2.5_, PM_10_, nitrogen dioxide (NO_2_)) and the risk of attention-deficit/hyperactivity disorder (ADHD) in children. Observational studies, including cohort, case-control, and cross-sectional designs, that examined the relationship between postnatal exposure to ambient air pollution and ADHD in children were included, while studies focusing on prenatal exposure or unrelated neurodevelopmental outcomes were excluded. A comprehensive search of databases including PubMed, Web of Science, Embase, and Ovid, last updated in May 2024, was conducted. The risk of bias in the selected studies was assessed using the Joanna Briggs Institute (JBI) critical appraisal tools, with discrepancies resolved through discussion among four reviewers. A meta-analysis was performed, synthesizing results using hazard ratios (HR), odds ratios (OR), and risk ratios (RR) as effect sizes. Random effects models were applied in most analyses due to the expected variability between studies, while fixed effects models were employed where only two studies were available. A total of 25 studies were included, with sample sizes ranging from 174 to 35,103 children. The studies were conducted in different countries and varied in their design and pollutant exposure measurement methods. The meta-analysis demonstrated a significant association between PM_2.5_ exposure and ADHD, with moderate heterogeneity (I² = 74.2%). PM_10_ exposure was also significantly associated with ADHD, and the heterogeneity was reduced to 34.94% after excluding an influential outlier. NO_2_ exposure similarly showed a significant association with ADHD, with low heterogeneity (I² = 0%). Due to the limited number of studies per pollutant (ranging from two to six), publication bias was not assessed. Despite the significant findings, there were limitations, including moderate to high heterogeneity among studies and the small number of studies per pollutant, which restricted the ability to assess publication bias and impacted the robustness of the results. Differences in exposure measurement methods and study designs also contributed to variability in the findings. Nonetheless, the evidence suggests that postnatal exposure to ambient air pollutants, particularly PM_2.5_, PM_10_, and NO_2_, is significantly associated with an increased risk of ADHD in children. These results underscore the importance of conducting further large-scale, high-quality studies to explore these associations in greater depth and to elucidate the mechanisms underlying the link between air pollution and ADHD.

## Introduction and background

Air pollution is expected to increase globally in the coming decades, driven by urbanization, industrialization, and rising energy demands. Current trends indicate that particulate matter (PM_2.5_, PM_10_) and nitrogen dioxide (NO_2_) levels remain high in rapidly developing regions, including parts of Asia and Africa, where population growth and increased transportation and industrial activity exacerbate air quality issues. Future projections suggest that, despite emission reduction strategies, pollutants like PM_2.5_ may continue to affect large populations due to changing climate conditions and more frequent extreme weather events, particularly in Asia, where the health burden is expected to intensify [1]. Studies show that climate change will likely exacerbate air pollution, with regions like China experiencing up to a 3-4% rise in PM_2.5_ and ozone levels by mid-century, increasing premature deaths due to pollution exposure [1,2]. Furthermore, if stringent climate policies are not enforced, the world could witness a significant rise in air pollution by 2050, with major urban centres facing worsened air quality [3]. These concerning trends are particularly relevant for vulnerable populations such as children, as growing evidence links air pollution to adverse neurodevelopmental outcomes, including an increased risk of attention-deficit/hyperactivity disorder (ADHD).

ADHD is a prevalent neurodevelopmental disorder characterized by persistent patterns of inattention, hyperactivity, and impulsivity. These symptoms often interfere with functioning or development and can persist into adulthood. The global prevalence of ADHD in children and adolescents ranges from 2% to 7%, with variations observed across different regions and populations [4]. In the United States, around 9.8% of children aged 3-17 years are diagnosed with ADHD, with higher rates observed in boys compared to girls. Europe reports prevalence rates between 3.3% and 7.8%, while Asia generally shows lower rates, ranging from 1.3% to 4.7%. In Africa, prevalence rates are about 1.5-5.4%, and in Latin America, they range from 5% to 10% [5-7]. Boys are more frequently diagnosed with ADHD than girls, with a ratio of approximately 4:1 in clinical samples and 2.4:1 in population studies [4]. ADHD often persists into adolescence and adulthood, leading to long-term impairments in educational achievement, employment, and social relationships, necessitating sustained public health efforts [8]. The high prevalence of comorbid mental health conditions among individuals with ADHD adds to the overall burden and complexity of the disorder, requiring comprehensive care strategies [9].

The aetiology of ADHD is multifactorial, involving genetic, environmental, and neurobiological factors. Among environmental factors, exposure to air pollutants has garnered significant attention due to its potential impact on neurodevelopmental outcomes. Prenatal exposures to risks such as maternal smoking, alcohol consumption, and stress increase the likelihood of the disorder [10]. Perinatal complications, including low birth weight and preterm birth, are also linked to higher ADHD risk [11]. Postnatal factors, such as exposure to environmental toxins like lead and pesticides, and early childhood adversity including abuse and neglect, further contribute to ADHD development [12,13].

Air pollution impacts neurodevelopment through mechanisms like neuroinflammation, oxidative stress, and direct toxicity. Fine particulate matter (PM_2.5_) and pollutants can trigger brain inflammation, disrupting normal development. These particles reach the brain via the olfactory bulb or by crossing the blood-brain barrier, activating microglial cells, which release inflammatory cytokines, leading to chronic neuroinflammation. This process may contribute to ADHD symptoms [14]. Oxidative stress is another key mechanism. Pollutants like PM_2.5_, NO_2_, and ozone generate reactive oxygen and nitrogen species, causing cellular damage. This oxidative stress can impair neurons, glial cells, and neurotransmitter function, contributing to ADHD-related cognitive and behavioural issues [15]. The evidence for ozone (O_3_) and sulphur dioxide (SO_2_) is weaker. While ozone can cause inflammation, its link to ADHD is less consistent, and limited research connects SO_2_ to ADHD [16]. Traffic-related air pollution and particulate matter exposure have been associated with higher odds of developing these conditions, likely due to the neurotoxic effects of pollutants on the developing brain [17,18].

Despite a growing body of research investigating the link between air pollution and the development of ADHD in children, substantial gaps and inconsistencies remain. These discrepancies arise from varying associations reported across pollutants like PM_2.5_ and NO_x_, as highlighted in studies by Zhao et al. [4] and Dalla et al. [19]. Such inconsistency could be due to differences in study designs, exposure assessments, and population characteristics, underscoring the need for a systematic review with stringent inclusion criteria to reduce variability and improve comparability. Furthermore, existing meta-analyses, such as those conducted by Donzelli et al. [20] encompass diverse methodologies and diagnostic criteria, adding to the heterogeneity in findings. Geographical and demographic disparities in studies, like those by Kaur et al. [21] and Aghaei et al. [17], limit the generalizability of the findings, suggesting the need for an expanded review that includes studies from various settings. Our systematic review and meta-analysis (SRMA) on the link between air pollution and ADHD development offers several distinctive features that set it apart from previous reviews on the subject. It serves as an updated synthesis of the latest research, incorporating studies up to the present date, which allows for a more current understanding of the trends and shifts in research focus. By including these methodological refinements and focusing on the latest available data, our SRMA aims to provide a more precise and up-to-date picture of how air pollution impacts ADHD in children.

## Review

Methodology

Protocol and Registration

This SRMA were conducted according to a protocol registered with the International Prospective Register of Systematic Reviews (PROSPERO) (registration number: CRD42022333585), adhering to the Preferred Reporting Items for Systematic Reviews and Meta-Analyses (PRISMA) guidelines for systematic reviews.

Inclusion and Exclusion Criteria

Included studies were observational (cross-sectional, case-control, and cohort) focusing on children exposed to ambient air pollution postnatally. Studies were selected based on their relevance to the research question, without specific restrictions on the publication date. Only studies that specifically measured the key pollutants mentioned below in the definition of ambient air pollution were considered for inclusion.

Articles were excluded if they addressed unrelated pollutants or medical conditions such as studies focusing on indoor air pollution, noise pollution, or outcomes unrelated to ADHD like cognitive performance or behavioural problems not specifically linked to ADHD. Additionally, studies that involved populations not relevant to our review such as adults or specific non-generalizable cohorts, were excluded. Articles that were clearly identified as reviews, letters to the editor, conference abstracts, controlled trials, case reports, interventional studies, and in vitro or animal studies, or that dealt with prenatal exposure scenarios, which were outside the scope of our review criteria were excluded. Lastly, articles written in languages other than English were also discarded.

Search Strategy

A comprehensive literature search was conducted across several databases (PubMed, Web of Science, Embase, and OvidSP) and clinical trial registries from the United States, Europe (including the United Kingdom), WHO, Australia, and India. The PubMed search strategy was developed using a process that included identifying three main concepts related to the research question: ambient air pollution, ADHD, and children. These terms were initially explored in Google Scholar to identify related keywords. Each keyword was then checked for its inclusion in the Medical Subject Headings (MeSH) database; if it was listed, it was added as a MeSH term, and if not, it was included as a keyword. The search involved applying truncation and quotation marks as needed and using Boolean operators to combine the terms effectively. The detailed PubMed search strategy retrieved a total of 290 articles. The comprehensive search strategy, categorized by database along with other supplementary files, can be found in the Open Science Framework (OSF) link provided in the Appendices.

Definition of Ambient Air Pollution

The study conformed to the World Health Organization’s definition of ambient air pollution [22], focusing on key pollutants known to impact air quality and public health. These pollutants include PM_10_ and PM_2.5_, carbon monoxide (CO), O_3_, NO_2_, and SO_2_. These substances were chosen due to their well-documented effects on human health, particularly concerning respiratory and neurodevelopmental outcomes in children.

ADHD Case Definition

The primary basis for ADHD diagnoses included in the review was the Diagnostic and Statistical Manual of Mental Disorders criteria, specifically the Fourth Edition (DSM-IV) [23] and Fifth Edition (DSM-V) [24]. In addition to clinical diagnoses, the review considered studies utilizing scale-based diagnostic tools. The parent ratings were taken as ADHD which involved the use of the Child Behavior Checklist (CBCL). Studies that included assessments by teachers, either alone or integrated with Conners' Teacher Rating Scales, were also considered. The diagnosis by neuropsychological tests was also included. These tests measured cognitive impairments like hit reaction time, omission and commission error directly associated with ADHD which ensured objective evaluation of the neurocognitive deficits that characterize the disorder.

Study Selection

The process of selecting studies for inclusion in our systematic review was systematic and followed a pre-defined protocol to ensure consistency and minimize bias. After the initial search, duplicates were removed using automated software tools followed by a manual check to ensure no relevant studies were inadvertently excluded.The remaining studies underwent a two-phase screening process:

Title and abstract screening: Each study's title and abstract were independently screened by two reviewers (PK, SA). This initial screening was based on explicit inclusion and exclusion criteria developed for this review. The inclusion criteria mandated that the studies be epidemiological in nature, written in English, and specifically investigate the relationship between exposure to ambient air pollutants (as defined by the WHO) and ADHD outcomes in children. During this phase of our systematic review, studies were meticulously evaluated to ensure alignment with the inclusion criteria. The primary reason for exclusion during title screening was studies not being directly relevant to the specific focus of our review on ambient air pollution and ADHD in children. Other exclusion criteria are given above in the relevant section.

Full-text review: Studies that passed the title and abstract screening were subjected to a full-text review, where the same two reviewers independently assessed the complete articles to confirm eligibility based on the detailed criteria. At both stages of the screening process, any discrepancies between the reviewers were initially discussed to reach a consensus. All the discrepancies were resolved through discussion.

Documentation and Workflow Management

All screening decisions, including reasons for excluding studies at the full-text review stage, were documented systematically. This documentation was maintained in a review manager software, Zotero (Corporation for Digital Scholarship, Vienna, Virginia, United States), which facilitated transparency and allowed for an audit of the selection process if necessary.

Data Collection Process

The data extraction form was designed to systematically gather key information from each study. It included details such as authors, publication year, and country, along with the study design and sample size. The form recorded participant demographics (age and sex), type of air pollution exposure, and measurement methods. It also listed confounders considered, ADHD outcomes and diagnostic tools used, key study conclusions, and reported odds ratios (OR) or relative risks (RR). In cases where full-text articles were not accessible, we contacted the corresponding authors of nine studies via email to request access. Of these, we successfully received responses and obtained full-text versions of four articles. There was no missing data in the final dataset used for the SRMA.

Risk of Bias Assessment

The risk of bias in the included studies was assessed using three specific Joanna Briggs Institute (JBI) critical appraisal tools tailored for different study designs: case-control, cohort, and cross-sectional studies [25] The JBI tool for case-control studies evaluated the similarity of groups, exposure measurement methods, and strategies for managing confounding factors. For cohort studies, the tool examined whether the groups were comparable, the validity and reliability of exposure measurements, and whether outcomes were measured appropriately over a sufficient follow-up period. The cross-sectional studies tool focused on the clarity of inclusion criteria, detailed descriptions of study subjects and settings, and the validity and reliability of both exposure and outcome measurements. The maximum possible JBI scores varied by study design: 11 for cohort studies, 10 for case-control studies, and 8 for cross-sectional studies. Despite these differences in maximum scores, we kept the classification criteria for all study types consistent. Studies scoring 8 or higher were categorized as high quality, those scoring between 5 and 7 were classified as moderate quality, and studies with a score of 4 or lower were categorized as low quality.

Each tool ensured a thorough evaluation of methodological quality and addressed potential biases, with independent assessments conducted by NKG and AMB. Discrepancies were resolved through discussion or consultation with a third reviewer (RR).

Statistical Analysis

The data extracted were entered in Excel (Microsoft Corporation, Redmond, Washington, United States). For data analysis, we used Jamovi version 2.3.28 version (https://www.jamovi.org).

Separate analyses were performed for each pollutant (PM_2.5_, PM_10_, NO_2_). Hazard ratios (HR), odds ratios (OR), and risk ratios (RR) were the effect sizes analyzed. Due to the low prevalence of ADHD, we combined OR and RR in a single analysis to enhance comparability across studies. A random effects model was employed for most analyses, based on the assumption that the true effect sizes varied between studies due to differences in populations, methodologies, and exposure measurement. However, in cases where only two studies were available, we applied a fixed effects model under the assumption that the effect size was homogeneous and that both studies estimated the same underlying effect.

The meta-analysis was based on several key assumptions. First, we assumed independence of studies, where each study provided an independent estimate of the effect size, with no overlap in participants. Additionally, we assumed that the effect sizes followed a normal distribution around the overall effect size, particularly in the random effects model. Publication bias assessments, such as funnel plots and Egger's test, are typically reliable when there are at least 10 studies included in the meta-analysis. With fewer than 10 studies, these tests are generally not recommended as they can lead to inaccurate or misleading results. Since our meta-analysis included between two to six studies for each pollutant, it was not appropriate to conduct publication bias assessments in this case [26].

We performed outlier and influential case diagnostics, identifying and removing studies with extreme effect sizes or those that exerted disproportionate influence on the pooled estimate. Sensitivity analyses were conducted to examine the impact of removing these studies on the overall results. We also quantified heterogeneity among studies using the I² statistic.

Results

**Figure 1:**
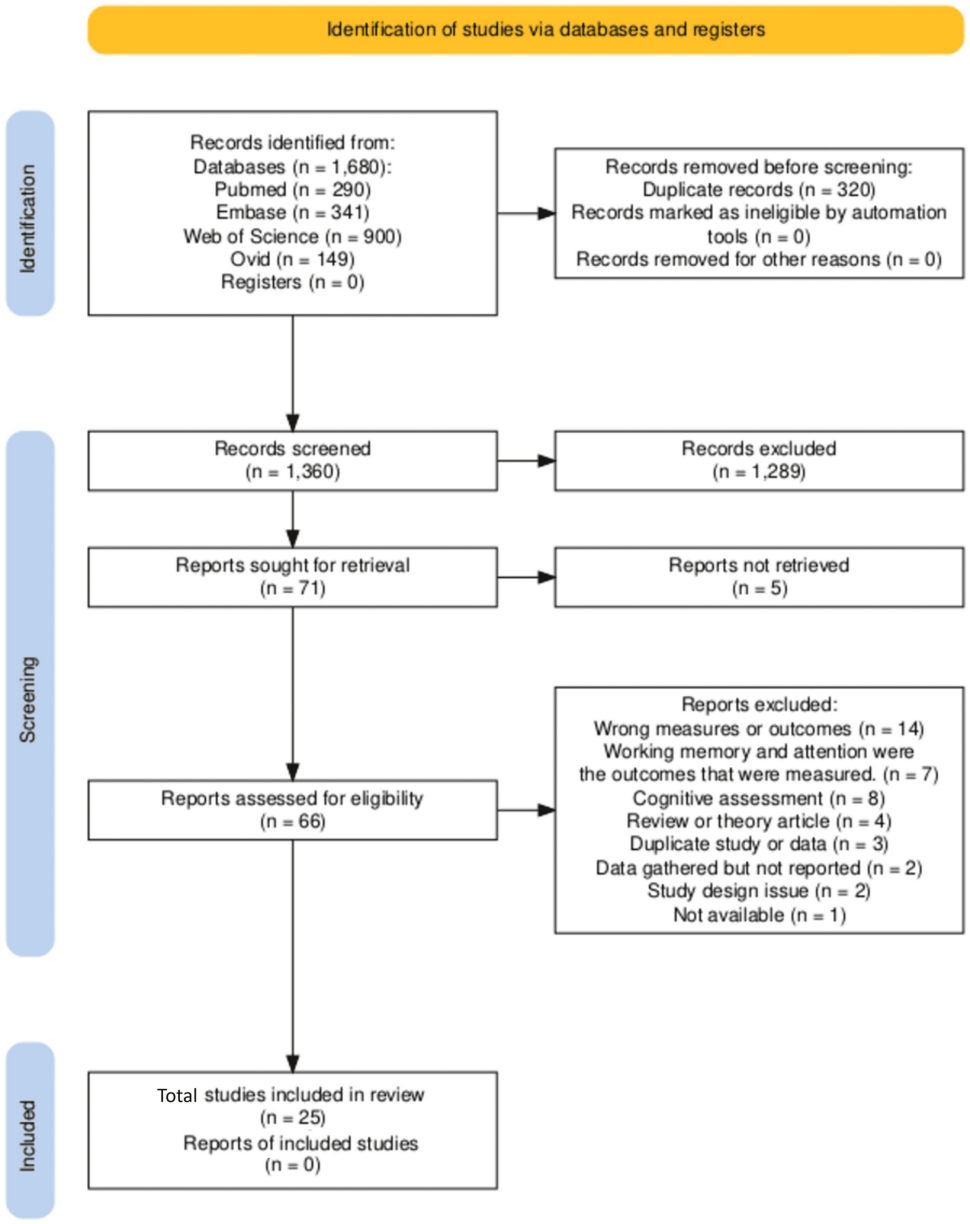
PRISMA flow diagram showing steps of study selection for the systematic review PRISMA: Preferred Reporting Items for Systematic Reviews and Meta-Analyses

The study selection process followed the PRISMA 2020 guidelines and is illustrated in the flow diagram (Figure 1) [27]. The initial search across five databases (PubMed, Embase, Web of Science, Ovid, and Registers) retrieved a total of 1,680 records. After removing 320 duplicate records, 1,360 studies remained for the screening process. During the title and abstract screening, 1,289 studies were excluded based on their irrelevance to the research focus on ambient air pollution and ADHD. Following this, 71 full-text reports were sought for retrieval, but five were not available for full-text review. Among the 66 full-text reports assessed for eligibility, 41 studies were excluded for reasons such as wrong measures or outcomes (n = 14), assessing working memory and attention rather than ADHD outcomes (n = 7), or utilizing cognitive assessments instead of ADHD-specific measures (n = 8). Other exclusions were due to duplicate data, study design issues, or unavailable data. Finally, 25 studies were included in the systematic review, with no additional reports of newly included studies.

The results of the risk of bias assessment using the JBI score are shown in Table 1. Among the cohort studies, 11 were classified as high quality, with scores ranging from 8 to 10, indicating strong methodological rigour. These included studies such as those by Alemany et al. [28], Chang et al. [29], and Min et al. [30], all scoring 9 or higher. The remaining cohort studies were classified as moderate quality, with scores ranging from 5 to 7, such as Fuertes et al. [31] and Rivas et al. [32]. In the cross-sectional studies, three were classified as high quality (Forns et al. [33], Zhang et al. [34], and Zhou et al. [35]) with a perfect score of 8, while one study (Siddique et al. [36]) was categorized as moderate quality, scoring 7. The single case-control study by Shim et al. [37] scored 9, classifying it as high quality.

**Table 1:** Assessment of risk of bias and study quality based on JBI scores JBI: Joanna Briggs Institute

Sl. No	Authors	JBI Score	Study design	Quality of study
1	Alemany et al. (2018) [28]	9	Cohort	High
2	Chang et al. (2022) [29]	9	Cohort	High
3	Chen et al. (2024) [38]	9	Cohort	High
4	Chiu et al. (2013) [39]	4	Cohort	Moderate
5	Fan et al. (2022) [40]	7	Cohort	Moderate
6	Forns et al. (2016) [33]	7	Cross sectional	Moderate
7	Forns et al. (2017) [41]	8	Cohort	Moderate
8	Fuertes et al. (2016) [31]	5	Cohort	Moderate
9	Gong et al. (2014) [42]	7	Cohort	Moderate
10	Markevych et al. (2018) [43]	8	Cohort	High
11	Liu et al. (2023) [44]	7	Cohort	Moderate
12	Loftus et al. (2020) [45]	9	Cohort	High
13	Min and Min (2017) [30]	10	Cohort	High
14	Newman et al. (2013) [46]	9	Cohort	High
15	Rivas et al. (2019) [32]	5	Cohort	Moderate
16	Roberts et al. (2019) [47]	9	Cohort	High
17	Sentís el al. (2017) [48]	9	cohort	High
18	Shim et al. (2022) [37]	9	Case Control	High
19	Siddique et al. (2011) [36]	7	Cross sectional	Moderate
20	Sunyer et al. (2017) [49]	7	Cohort	Moderate
21	Thygesen et al. (2019) [50]	9	Cohort	High
22	Yuchi et al. (2022) [51]	8	Cohort	High
23	Li et al. (2023). (2023) [52]	8	Cohort	High
24	Zhang et al. (2022) [34]	8	Cross sectional	High
25	Zhou et al. (2023) [35]	8	Cross sectional	High

The study characteristics, exposure to pollutants, and confounders of the included studies are given in Table 2. The study outcomes, measurements, and key findings of the included studies are given in Table 3.

**Table 2:** Study characteristics, exposure, and confounders of included studies EC: elemental carbon; NO_2_: nitrogen dioxide; APOE: Apolipoprotein E; PM: particulate matter; BC: black carbon; TRAP: traffic-related air pollution; UFP: ultrafine particles; NDVI: Normalized Difference Vegetation Index; MODIS: moderate-resolution imaging spectroradiometer; BMI: body mass index; KIDI: Knowledge of Infant Development Index; ECAT: elemental carbon attribuable to traffic; SDQ: Strengths and Difficulties Questionnaire; CAN: Canadian Marginalization Index

Sl. No	Authors (Year)	Country	Study Design	Sample Size	Age Range	Pollutant/Exposure	Exposure Measurement	Confounders
1	Alemany et al. (2018) [28]	Spain	Prospective cohort study	2,897	7-11 years	EC, NO_2_	EC and NO_2_ levels were measured using land use regression models based on two 1-week campaigns conducted six months apart in 2012. The study estimated yearly outdoor air pollution levels for each pollutant based on these measurements.	Age, APOE ε⁢4 status, birth weight, duration of breastfeeding, educational level of parents, exposure to environmental tobacco smoke, gender, genetic factors, maternal smoking, number of siblings, school performance, socioeconomic status, and vulnerability index
2	Chang et al. (2022) [29]	Taiwan	Prospective cohort study	425,736	0-5 years	PM_2.5_	Daily PM_2.5_ concentrations derived from a 1-km satellite-based estimation model. Measured during the first, second, and third trimesters and from age 1–5 years after birth.	Child comorbidities (e.g., asthma, allergic rhinitis), chronic diabetes disease, chronic hypertension, drug use, gestational diabetes mellitus, gestational hypertension, heart diseases, infant birth weight, iron deficiency anemia, maternal age at delivery, maternal anemia, maternal smoke, polyhydramnios and oligohydramnios, preeclampsia, preterm birth, socioeconomic status, and sex.
3	Chen et al. (2024) [38]	Spain	Prospective cohort study	1,416	Early childhood (Assessments around ages 5 and 7)	PM_2.5_	Daily residential PM_2.5_ exposures were estimated using a two-stage random forest model with temporal back-extrapolation, averaged over 1-week periods in the prenatal period and 4-week periods in the postnatal period.	Child age, child sex, exclusive formula feeding, maternal education, maternal verbal intelligence quotient (IQ score), parity, season, self-reported breastfeeding, smoking during pregnancy, and social class.
4	Chiu et al. (2013) [39]	United States	Prospective cohort study	174	7-14 years	BC	BC levels estimated using a validated spatiotemporal land-use regression model based on children's residences throughout their lifetimes.	Age, blood lead levels, child IQ, community- level social stress, maternal education, pre- and postnatal tobacco smoke exposure, and sex.
5	Fan et al. (2022) [40]	Taiwan	Retrospective cohort study	98,177	Under 18 years	PM_10_, PM_2.5_	Daily PM_2.5_ and PM_10_ concentrations sourced from the Taiwan Air Quality Monitoring Database. Exposure assessments were based on data from air quality monitoring stations located in the participants' living areas.	Comorbid conditions (asthma, allergic rhinitis, atopic dermatitis), sex, socioeconomic factors, and urbanization level
6	Forns et al. (2016) [33]	Spain	Cross-sectional study	2,897	7-11 years	EC, BC, NO_2_	EC, BC, and NO_2_ were measured at schools in two 1-week campaigns six months apart, both indoors and outdoors. EC levels were determined by chemical analysis of PM_2.5_ samples, and real- time BC concentrations were measured with a MicroAeth AE51 aerosol monitor. Weekly averaged NO_2_ concentrations were obtained using Gradko Environmental passive dosimeters.	Child's age, maternal education, sex, socioeconomic status, tobacco smoke exposure at home, traffic noise annoyance at home, and urban vulnerability index of the home and school.
7	Forns et al. (2017) [41]	Spain	Prospective cohort study	2,897	7-12 years at the start, followed over 3.5 years	EC, NO_2_, PM_2.5_, UFP	Measured at 39 schools across Barcelona, using real-time concentrations for UFP and BC during class time. TRAPs (EC, NO_2_, PM_2.5_) were measured indoors and outdoors at schools during two separate one-week campaigns in different seasons.	Age, child's sex, green spaces at schools, home air pollution exposure (NO_2_), maternal education, noise, socioeconomic status, and Urban Vulnerability Index.
8	Fuertes et al. (2016) [31]	Germany	Prospective cohort study	4,745	0-15 years	NO_2_, PM_10_, PM_2.5_, EC	Annual average concentrations at participants' birth, 10 years, and 15 years addresses estimated using land-use regression models.	Age at follow-up, cohort/intervention group, distance to green spaces, maternal age at birth, maternal smoking during pregnancy, outdoor time, parental education, parental psychopathology, screen time, secondhand smoke exposure, sex, and single parent status.
9	Gong et al. (2014) [42]	Sweden	Prospective cohort study	3,426	9-12 years	PM_10_, NO_2_	Exposure to PM_10_ and NO_2_ was estimated at participants’ addresses during pregnancy, the first year, and the ninth year of life using dispersion modeling, controlling for seasonal variation.	Birth weight, family disposable income, gender, gestational age, maternal age at birth, maternal marital status, maternal smoking during pregnancy, parental education, parity, Socio-economic status, and neighborhood deprivation index.
10	Markevych et al. (2018) [43]	Germany	Retrospective cohort study	66,823	0-14 years	PM_10_, NO_2_	Annual average concentrations of PM_10_ and NO_2_ for the year 2007 derived from land use regression models; NDVI assessed from MODIS satellite images over 2005-2014 to represent vegetation levels.	Child/adolescent psychiatrists, healthcare access, population density in postal code areas, proximity to healthcare providers, sex, socio-economic status indicators (like long- term and overall unemployment), and year of birth.
11	Liu et al. (2023) [44]	China	Cross-sectional study	164,081	6-18 years	PM_2.5_	Satellite based random forest approach at a spatial resolution of 1 KM which included ground monitored data, satellite remote sensing and land use.	Annual average household income, birth weight, BMI, breastfeeding, cesarean section, construction/renovation activities, exposure to secondhand tobacco, gender, outdoor physical exercise, parental education, pet ownership, prematurity, and residential proximity to roads with heavy traffic.
12	Loftus et al. (2020) [45]	United States	Prospective cohort study	975	1.5- 5 years	PM_10_, NO_2_	Prenatal and postnatal exposures estimated at the children's residences using a universal kriging model incorporating land-use regression with spatial smoothing, based on national air quality monitoring data and various geographic covariates.	Age of mother, birth order, breastfeeding, child sleep score at assessment, education per-natal smoking, individual- and neighborhood-level socioeconomic measures, insurance status, KIDI Score, maternal age, maternal BMI, maternal IQ, Neighborhood COI, paternal education, prenatal nutrition (maternal plasma folate), prenatal vitamin use, pre-natal depression, race, and other potential confounders.
13	Min and Min (2017) [30]	South Korea	Retrospective cohort study	8,936	0-10 years	PM_10_, NO_2_	Annual mean levels of PM_10 _and NO_2_ were assessed from birth to diagnosis using National Ambient Air Monitoring System data interpolated for each participant's district code using geographic information systems (GIS) with Universal Kriging technique.	Gender, history of diseases (including meningitis, iron deficiency anemia, thyroid disorder), household income, and metropolitan area residence.
14	Newman et al. (2013) [46]	USA	Prospective cohort study	762	Assessed at 7 years of age	EC	ECAT exposure during the first year of life was estimated using land-use regression modeling based on 27 air sampling sites around Cincinnati. The modeling considered traffic volume and distance to major highways.	Age of home (surrogate for lead exposure), cotinine measurement, duration of breastfeeding, environmental tobacco smoke, genetic predispositions, household income, indoor environmental factors (e.g., tobacco smoke), insurance status, maternal education, parental report of child spending time at babysitter or relatives' home, paternal education, proximity to major highways, race, socioeconomic status, and ethnicity.
15	Rivas et al. (2019) [32]	Spain	Prospective cohort study	2,221	7-10 years	PM_2.5_	Land use regression models for both the prenatal period and the first seven postnatal years.	Age, sex, parental education, occupation, marital status, family origin, residence history, maternal education, Urban Vulnerability Index, parental education level, exposure to environmental tobacco smoke, SDQ scores, and other sociodemographic factors
16	Roberts et al. (2019) [47]	United Kingdom	Prospective cohort study	284	Assessed at ages 12 and 18	PM_2.5_, NO_2_	High-resolution annualized average concentration estimates based on pollution measurements from several sources, modeled at a resolution of 20m x 20m around the participants' addresses.	Ethnicity, exposure to severe childhood victimization, family psychiatric history, family SES, neighborhood socioeconomic status, sex, and smoking.
17	Sentís el al. (2017) [48]	Spain	Prospective cohort study	1,298	4-5 years	NO_2_	Estimated prenatal and postnatal NO_2_ levels at the participants' residential addresses using land-use regression models.	Exposure to environmental tobacco smoke, household gas appliance, maternal alcohol use, maternal consumption of fish, fruits, vegetables, vitamin D, and folic acid, maternal education, maternal height and pre- pregnancy weight, maternal IQ, maternal noise annoyance, maternal smoking and exposure to secondhand smoke, maternal and paternal countries of birth, parental age, paternal BMI, socioeconomic status, and urbanicity.
18	Shim et al. (2022) [37]	South Korea	Cross-sectional study	1,120	6-19 years	PM_10_	PM_10_ concentrations were assessed annually using data from national air quality monitoring stations matched to participants' administrative district codes for the year of diagnosis.	Absence due to illness, age, basic livelihood security, body mass index , family income, food security, heavy drinking, parental stress, region (urban/rural), secondhand smoke exposure at home, self-reported health status, sex, and type of health insurance.
19	Siddique et al. (2011) [36]	India	Cross-sectional study	969 urban 850 rural	9-17 years	PM_10_, SO_2_, NO_2_	PM_10_ levels were obtained from Central Pollution Control Board and aerosol monitors. Measurements were taken in the urban environment of Delhi, where levels significantly exceeded those of rural control areas.	Age group, BMI, environmental tobacco smoke, gender, indoor air pollution from household fuel use, parental smoking, and socioeconomic status.
20	Sunyer et al. (2017) [49]	Spain	Prospective cohort study	2,687	7-10 years	NO_2_, EC, PM_2.5_	Daily ambient levels of NO_2_ and EC measured at a fixed air quality background monitoring station and in schools. NO_2_ real-time chemiluminescence using model SIR 5012 EC using PM_2.5_ filters	Age, air pollution at home, maternal education, school achievement, school noise, sex, socioeconomic status of the neighborhood, temperature, time of day, and various environmental and contextual variables.
21	Thygesen et al. (2019) [50]	Denmark	Prospective cohort study	809,654	0-21 years	NO_2_, PM_2.5_	Exposure levels were measured using a high-resolution THOR model to estimate daily concentrations of NO_2_ and PM_2.5_ at a 1 km x 1 km resolution at residences from birth to the fifth birthday.	Age, income, parental education, sex, and year of birth.
22	Yuchi et al. (2022) [51]	Canada	Prospective cohort study	37,000	0-10 years	PM_2.5_, NO_2_	PM_2.5_ and NO_2_ estimated using land use regression models; noise estimated using a deterministic model; greenness estimated using vegetation percentage derived from linear spectral unmixing of Landsat imagery. Exposure period was from birth until the age of three.	Birth weight, CAN-Marg index, education, gestational age, household income, infant sex, maternal age, maternal BMI, maternal parity, paternal age, and season of birth.
23	Li et al.(2023) [52]	Netherlands	Prospective cohort study	2,750	10-12 years at start, followed up to ages 24-28	PM_2.5_, PM_10_, O_3_, SO_2_, NO_2_	Exposure was assessed at the residential level using standardized protocols and included longitudinal data on ambient noise and air pollution.	Psychostimulant medication, lifetime parental psychopathology, maternal smoking during pregnancy, parental socioeconomic status, and problematic pregnancy.
24	Zhang et al. (2022) [34]	USA	Cross-sectional study	235	6-14 years	PM_10_	PM_10 _measured inside children’s homes using personal modular impactors.	Age, ethnicity, neighborhood poverty, sex, and traffic proximity.
25	Zhou et al. (2023) [35]	China	Cross-sectional study	35,103	3-12 years	Ozone	Measured by the 4-year ambient ozone exposure level, collected from 2009 to 2012, using a random forest model to simulate high-resolution ozone concentration.	Age, gender, BMI, birth weight, premature delivery, breastfeeding status, exercise time per week, parental education level, household income, maternal alcohol consumption during pregnancy, early-life cigarette exposure, home renovation, and season.

**Table 3:** Study outcomes, measurements, and key findings SDQ: Strengths and Difficulties Questionnaire; ADHD: attention-deficit/hyperactivity disorder; DSM: Diagnostic and Statistical Manual of Mental Disorders; EC: elemental carbon; NO_2_: nitrogen dioxide; ICD=International Classification of Diseases; CM: clinical modification; PM: particulate Matter; HR: hazard ratio; RRcum: aggregated risk ratio; βcum: aggregated effect estimate; BC: black carbon; HRT: hit reaction time; aHR: adjusted hazard ratio; TRAP: traffic-related air pollution; IQR: interquartile range; UFP: ultrafine particles; ASD: autistic spectrum disorders; CBCL: Child Behavior Checklist; NDVI: Normalized Difference Vegetation Index; SES: socioeconomic status; TR: text revision; SE: standard error; ECAT: elemental carbon attribuable to traffic; DCR: diagnostic criteria for research; K-CPT: Kiddie-Conners Continuous Performance Test; IRR: incidence rate ratio; aHR: ajusted hazard ratios; SO_2_: sulphur dioxide; ADHD-T: attention-deficit/hyperactivity disorder tendencies; ADHP: attention-deficit/hyperactivity problems; DSM-IV: Diagnostic and Statistical Manual of Mental Disorders, Fourth Edition; aMR: adjusted mean ratios; A-TAC: Autism-Tics, ADHD, and other Comorbidities Inventory

Sl No	Authors (Year)	Outcome Measurement	Key Conclusions	Results
1	Alemany et al. (2018) [28]	Behavior problems: SDQ total difficulties score ADHD symptoms: ADHD-DSM-IV questionnaire score Cognitive function: Repeated computerized tests of inattentiveness and working memory	Exposure to EC, and NO_2_ was associated with higher behavior problem scores and smaller reductions in inattentiveness over time.	Increases in air pollution levels were significantly associated with behavior problems β = 0.08 (95% CI: 0.03, 0.13) per IQR increase in NO_2_. ADHD Symptom score (mean ratio) EC 0.97 (95% CI: 0.90,1.04), NO_2_ 1.05 (95% CI:0.95, 1.16). Inattentiveness trajectories (β) EC 2.48 (95% CI: -1.08, 6.05), NO_2_ 2.22 (95% CI: -2.68, 7.12)
2	Chang et al. (2022) [29]	ADHD diagnosed using ICD-9-CM code 314 in inpatient and outpatient records.	Incidence of ADHD was 2.2%. Exposure to PM_2.5_ during the prenatal and early postnatal periods is associated with an increased risk of ADHD. The risk increases with higher PM_2.5_ levels, particularly during the first trimester and early years of life.	Year 1-3 of Life HR: Ranged between 1.40 and 1.87 per 10 μg/m³ increase in PM_2.5_ - Dose-Response Relationship: ADHD risk sharply increased with PM_2.5_ >50 μg/m³ during the year 3 of life. HR at fifth year of life: 0.30 per 10 μg/m³ increase (95% CI: 0.16, 0.57), HR at third year of life: 1.87 per 10 μg/m³ increase (95% CI: 1.53, 2.29), HR in Boys 1.87 (95% CI:1.48, 2.37), HR in Girls 1.85 (95% CI: 1.24,2.76)
3	Chen et al. (2024) [38]	At around 5 years of age, teachers reported ADHD symptoms using the ADHD DSM-IV. At around 7 years of age, parents completed the Conners' Parent Rating Scales, evaluating the ADHD index, cognitive problems/inattention, hyperactivity, and oppositional subscales.	Median age 4.5 years. Exposure to PM_2.5_ during specific windows in mid-gestation and early toddlerhood is associated with increased hyperactivity symptoms at ages 5 and 7.	Exposure associated with increased hyperactivity subscale around age 7 (βcum = 3.70, 95% CI = 2.36, 5.03). PM_2.5_ RRcum 2.78 (95% CI: 2.02-3.82) in age range 1.2- 2.9 years PM_2.5_ RRcum 0.81 (95% CI: 0.74-0.89) in age range 3.8- 4.1 years PM2.5 βcum 4.19 (95% CI: 2.78-5.61) in age range 0.9- 2.7 years for Tscore for the hyperactivity subscale.
4	Chiu et al. (2013) [39]	The Conner’s Continuous Performance Test (CPT) was used, where: Omission errors measure failure to respond to targets. Commission errors measure responses to non-targets. HRT measures the reaction time. Higher scores indicate increased errors or slower reaction time.	Increased levels of BC were associated with higher commission errors and slower HRT in children, particularly boys.	Commission Errors: Increased errors associated with higher BC levels; more pronounced in boys (β = 8.88, (95% CI: 2.64, 15.1) for 2nd quartile of BC; β = 9.17 (95% CI: 1.54, 16.8 for 3rd quartile). HRT: Slower times with higher BC; also, more pronounced in boys (β = 10.1, 95% CI: 0.42, 19.8 for 2nd quartile of BC, β = 7.9, 95% CI: -6.24, 16.8 for 4th quartile of BC). Commission error beta value on Multivariate Linear Regression: 2nd quartile 6.15 (95% CI: 2.03-10.27), 3rd quartile 4.75 (95% CI: 0.36, 9.14), 4th quartile 3.32 (95% CI: -0.87, 7.51) HRT beta value on Multivariate Linear Regression: 2nd quartile 6.51 (95% CI: 0.43-12.59) 3rd quartile 4.75 (95% CI: 0.36, 9.14), 4th quartile 3.32 (95% CI: -0.87, 7.51)
5	Fan et al. (2022)[40]	ADHD diagnosis based on having two or more outpatient diagnoses or one admission record, coded according to the ICD-9 and ICD-10.	Mean Age 9.6 years. Incidence of ADHD was 2.9%. Exposure to higher levels of PM_2.5_ and PM_10_ during early childhood is significantly associated with an increased risk of ADHD. The risk is dose-dependent, with higher pollution levels correlating with a greater risk of developing ADHD.	PM_2.5_: aHR = 1.79 (95% CI: 1.58–2.02) for the highest quartile of exposure compared to the lowest. PM_10_: aHR = 1.53 (95% CI: 1.37–1.70) for the highest quartile. The risk increased progressively with higher quartiles of PM_10_ and PM_2.5_ concentrations. PM_2.5_ aHR : 2nd quartile (Q2): aHR 0.20 (95% CI: 0.15-0.26), 3rd quartile (Q3): aHR 1.90 (95% CI: 1.70-2.13), 4th quartile (Q4): aHR 1.79 (95% CI: 1.58-2.02), PM_10 _aHR 2nd quartile (Q2): aHR 0.95 (95% CI: 0.84-1.07), 3rd quartile (Q3): aHR 2.02 (95% CI: 1.82-2.25), 4th quartile (Q4): aHR 1.53 (95% CI: 1.37-1.70))
6	Forns et al. (2016) [33]	General behavioral development was assessed using the SDQ filled out by parents. Specific ADHD symptoms were reported by teachers using the ADHD criteria of the DSM-IV.	Mean age 8.55 years. Exposure to TRAPs (EC, BC, NO_2_) and noise at school were associated with increased behavioral problems and ADHD symptoms among schoolchildren. TRAPs were particularly associated with higher SDQ total difficulties scores.	EC, BC, and NO_2_: Positive associations with SDQ total difficulties scores for IQR increases in indoor and outdoor levels (aMR around 1.07 for EC and NO_2_). Noise: Positive associations with ADHD-DSM-IV scores, indicating increased ADHD symptoms (aMR = 1.22 for an IQR increase in indoor noise levels). Statistical significance varied between single and multi-exposure models. Outdoor EC (IQR=0.86 μg/m3) aMR of SDQ Score increase by 1.07 (95% CI: 1.03-1.12). Outdoor EC (IQR=0.86 μg/m3) aMR of ADHD DSM-IV Score increase by 0.99 (95% CI: 0.93-1.07). Outdoor NO_2_ (IQR=22.3 μg/m3) aMR of SDQ Score increase by 1.07 (95% CI: 1.01-1.14). Outdoor NO_2_ (IQR=22.3 μg/m3) aMR of ADHD DSM-IV Score increase by 1.03 (95% CI: 0.94-1.13).
7	Forns et al. (2017) [41]	Working memory was evaluated using computerized n-back tests (3-back d′ as main outcome) conducted four times during the initial year and once more in 2015. Inattentiveness was assessed through sustained attention tasks.	Persistent exposure to TRAPs at school was negatively associated with cognitive development, particularly working memory, over a period of 3.5 years. The study suggests that detrimental impacts on cognitive development due to air pollution exposure at schools may have long-term effects.	NO_2_ Outdoor: Coefficient = -4.22, (95% CI: - 6.22, -2.22) Indoor UFP: Coefficient = -4.12 (95% CI: -5.68, -1.83). These coefficients represent the change in the annual rate of development in working memory for an interquartile range increase in TRAP exposure.
8	Fuertes et al. (2016) [31]	Hyperactivity/inattention assessed using the SDQ at 10 and 15 years. Dyslexia and dyscalculia reported by parents at 10 and 15 years.	Prevalence of ADHD was 12.9%. Exposure to PM_2.5_ and PM_2.5_ absorbance at 10- and 15-year addresses was associated with increased risk of hyperactivity/inattention.	Hyperactivity/inattention associated with PM_2.5_ at 10 years (OR = 1.12, 95% CI [1.01, 1.23]) and at 15 years (OR = 1.11, 95% CI [1.01, 1.22]) PM_10_ at 10 years (OR = 1.05, 95% CI [0.95, 1.17]) and at 15 years (OR = 1.02, 95% CI [0.93, 1.11])
9	Gong et al. (2014) [42]	ASD and ADHD were assessed using the A-TAC, which is a comprehensive screening tool based on DSM-IV criteria.	The study found no support for the hypothesis that exposure to traffic-related air pollution (NO_x_ and PM_10_) during pregnancy, the first year of life, or the ninth year of life is associated with ASD or ADHD in children.	NO_2_ at 9 years unadjusted OR 0.94 [0.50, 1.76]; aOR 1.15 [0.62-2.15]. PM_10_ at 9 years unadjusted OR 1.12 [0.57, 2.20]; aOR 1.33 [0.73-2.41]
10	Markevych et al. (2018) [43]	ADHD diagnosis based on ICD-10 criteria requiring age-inappropriate levels of hyperactivity and inattention that occur in two or more settings, present for at least six months, and not explained by other mental diseases.	Incidence of ADHD was 3.1%. Increased levels of PM_10_ and NO_2_ were associated with a higher risk of ADHD; exposure to higher greenspace (higher NDVI values) was associated with a reduced risk of ADHD.	PM_10_: An increase of 10 μg/m^3^ raised the relative risk of ADHD by 1.97 (95% CI: 1.35–2.86). NO_2_: An increase of 10 μg/m^3^ raised the relative risk of ADHD by 1.32 (95% CI: 1.10–1.58). NDVI: A 0.1-unit increase decreased the relative risk of ADHD by 0.82 (95% CI: 0.68–0.98).
11	Liu et al. (2023) [44]	DSM-IV checklist completed by parent or guardian	Mean age 11.7 years, Incidence of ADHD was 3.9%. The study found that long-term exposure to higher levels of ambient particulate matter (PM_1 _ and PM_2.5_) is significantly associated with increased odds of ADHD in school-aged children in China, emphasizing the critical importance of ambient PM concentration, size, components, and sources in addressing children's neurological health and designing effective interventions.	PM_1 _1.74 (1.47, 2.06) per 10 μg/m^3^, PM_2.5_ 1.65 (1.45, 1.88) per 10 μg/m3^3^, PM_1 _ Boys 1.74 (1.44, 2.10) per 10 μg/m^3^, PM_1 _Girls 1.72 (1.46, 2.03) per 10 μg/m^3^, PM_2.5_ Boys 1.65 (1.43, 1.91) per 10 μg/m^3^ PM_2.5_, Girls 1.61 (1.42, 1.83) per 10 μg/m^3^
12	Loftus et al. (2020) [45]	Assessed using the CBCL, focusing on internalizing and externalizing broad behavior problem domains.	4.3% of children reported externalizing behaviour above threshold value. Prenatal and postnatal exposure to NO_2_ is associated with an increase in externalizing behaviors. The effects of air pollution exposure are particularly pronounced in children from low SES backgrounds, suggesting increased vulnerability in these populations. PM_10_ and road proximity showed no association with outcomes.	Associations were stronger with postnatal NO_2_, particularly in children from lower SES families. OR postnatal for NO_2_ exposure for clinically significant externalizing problem 1.96 (1.03, 3.71) OR postnatal for PM_10_ exposure for clinically significant externalizing problem 0.87 (0.46, 1.64)
13	Min and Min (2017) [30]	ADHD diagnosed based on ICD-10 code F90.0 and DSM-IV-TR (corresponds to F90.0)	Incidence of ADHD was 3.5%. The study found significant associations between increased exposure to PM_10_ and NO_2_ from birth to diagnosis and higher incidence of ADHD among children, with exposure-response relationships indicating higher risks at higher pollutant tertiles.	PM_10_: HR = 1.17 (95% CI: 1.14-1.20) per 1 μg/m³ increase; in the highest tertile, HR = 3.73 (95% CI: 2.76- 5.03). NO_2_: HR = 1.03 (95% CI: 1.02-1.04) per 1 μg/m³ increase; in the highest tertile, HR = 2.16 (95% CI: 1.61- 2.90).
14	Newman et al. (2013) [46]	Behavioral Assessment System for Children, 2nd Edition (BASC-2) used at age 7. This tool assesses both adaptive and problematic behaviors in community and home settings. Scores were analyzed for hyperactivity, attention problems, aggression, conduct problems, and atypical behaviors.	Early-life exposure to higher levels of ECAT is significantly associated with increased risk of hyperactivity at 7 years of age. The effect is more pronounced in children from higher educational backgrounds of mothers.	Children exposed to the highest tertile of ECAT during the first year of life showed significantly higher hyperactivity scores compared to those with lower exposure. Adjusted odds ratio (aOR) for hyperactivity at 7 years of age was 1.7 (95% CI: 1.0, 2.7) for those in the highest exposure tertile. Hyperactivity unadjusted 1.9 (1.2, 2.9) adjusted 1.7 (1.0, 2.7) Attention problem unadjusted 1.4 (0.9, 2.2), adjusted 1.1 (0.6, 1.7)
15	Rivas et al. (2019) [32]	Working memory and attentiveness were assessed using computerized tests (n-back test for working memory and Attentional Network Test for attentiveness). The conflict network performance was evaluated based on reaction times in these tests.	Mean age 8.5 years. Exposure to PM_2.5_ during the fifth and sixth postnatal years was negatively associated with working memory performance. Higher PM_2.5_ levels during the prenatal period and from the fourth postnatal year were linked with reduced performance in conflict network, but not attentiveness.	The cumulative effect of a 10 µg/m³ increase in PM_2.5_ resulted in a reduction in working memory score by -19.50 points and an increase in the conflict attentional network time by 11.31 milliseconds, indicating poorer performance. The cumulative effect of a 10 µg/m³ increase in PM_2.5_ until 7th years of life resulted in reduction of attentiveness, HRT-SE (ms) beta= 5.29 (-18.1,7.49) Male = 10.26 (-27.69, 7.17), Female = 2.55 (-15.66, 20.75)
16	Roberts et al. (2019) [47]	Symptoms of mental health problems were assessed at ages 12 and 18 using standardized interviews and psychiatric assessments based on DSM criteria. ADHD symptoms at age 12 were ascertained via by mothers' and teachers' reports of inattention and hyperactivity as per DSM-IV and Rutter child scale. At age 18 DSM-IV and DSM-V criteria	Incidence of ADHD at age 18 was 5.6%. The study found no associations between air pollution exposure at age 12 and concurrent mental health problems. However, age-12 pollution estimates were significantly associated with increased odds of major depressive disorder at age 18, even after controlling for common risk factors.	Exposure to PM_2.5_ and NO_2_ at age 12 was associated with increased odds of depression at age 18 in both basic and full models (e.g., PM_2.5_ basic model OR = 1.69, 95% CI: 1.13–2.53; full model OR = 1.63, 95% CI: 1.08–2.46). No significant associations were found with anxiety or ADHD outcomes. OR for ADHD for PM_2.5_ Full model 1.16 (0.64, 2.10) and NO_2 _1.2 (0.69, 2.09)
17	Sentís el al. (2017) [48]	K-CPT outcomes included the number of omission errors (failure to respond to targets), commission errors (responses to non-targets), hit reaction time (time to respond to targets), and variability in response time (standard error of HRT).	Higher prenatal and postnatal exposure to NO_2_ is associated with impaired attentional function in children at 4-5 years of age, particularly affecting the standard error of hit reaction time and increasing omission errors.	Increase of 1.12 ms in HRT-SE per 10 µg/m³ increase in prenatal NO_2_ and a 6% increase in omission errors. Postnatal exposure results were similar but showed borderline significant increases in omission errors. Overall: HRT-SE beta 0.81 (-0.82, 2.43); Omission IRR 1.05 (0.99, 1.11); Commission IRR 1.04 (0.96, 1.13) Girls: HRT-SE beta 0.34 (-0.80, 1.49); Omission IRR 1.08 (1.00,1.17); Commission IRR 0.99 (0.94-1.04) Boys: HRT-SE beta 0.09 (-2.38, 2.55); Omission IRR 0.96 (0.85,1.10); Commission IRR 1.03 (0.94,1.14)
18	Shim et al. (2022) [37]	ADHD diagnosis was based on parental reports of ever having a doctor-diagnosed ADHD	The study found a significant association between PM_10_ exposure and the likelihood of ADHD diagnosis in children and adolescents, with higher PM_10_ concentrations linked to increased ADHD risk.	For each 10 µg/m³ increase in PM_10_: OR = 1.44 (95% CI: 1.02–2.02). The association was stronger at higher quartiles of exposure compared to lower quartiles, indicating a dose- response relationship, although not all comparisons reached statistical significance. OR for Male 1.35 (0.92, 1.98) and Female 2.17 (1.00, 4.72)
19	Siddique et al. (2011) [36]	ADHD symptoms assessed using a structured questionnaire completed by parents and teachers, evaluating inattentiveness, hyperactivity, and impulsivity.	Urban children in Delhi, exposed to higher levels of PM_10_, had a significantly higher prevalence of ADHD (11.0%) compared to rural children (2.7%). The study suggests a strong correlation between high PM_10_ levels and increased ADHD prevalence, controlled for potential confounders.	ADHD was significantly more prevalent in urban areas with high PM_10_ exposure, with an odds ratio (OR) of 2.07 (95% CI: 1.08-3.99), indicating a more than double risk compared to children with lower exposure levels. The highest ADHD prevalence was noted among boys and in the 12-14 age group. Male 1.5 (1.0, 2.3) PM_10_ 120-139 μg/m3 = 1.8 (1.1, 3.6) 140-200 μg/m3= 2.2 (1.2, 5.0) > 200 μg/m3 = 2.7 (1.4, 5.5)
20	Sunyer et al. (2017) [49]	Attention processes measured every three months over four repeated visits using the Child Attention Network Test (ANT). Working memory assessed using the n-back task.	Daily ambient levels of NO_2 _and EC were negatively associated with all attention processes, suggesting a short-term impact of air pollution on neurodevelopment. The study highlights that acute exposure to traffic-related air pollution can lead to fluctuations in attention among school children.	Children in the bottom quartile of daily exposure to ambient NO_2 _ had a 14.8 millisecond faster response time than those in the top quartile, which is equivalent to a 1.1- month retardation in natural developmental improvement in response speed with age. Adjustments for indoor levels of pollutants showed similar findings for EC.
21	Thygesen et al. (2019) [50]	ADHD diagnosis was identified using the International Classification of Diseases, 10th Revision, Diagnostic Criteria for Research (ICD- 10-DCR) codes F90x or F98.8.	Incidence of ADHD was 2.4%. Early childhood exposure to NO_2 _ and PM_2.5_ was associated with an increased risk of developing ADHD. The risk increases with higher exposure levels, even after adjusting for several confounders. NO2 showed a particularly strong association, even when controlled for PM2.5 exposure.	NO_2 _: IRR of 1.38 (95% CI: 1.35 to 1.42) per 10 µg/m³ increase. PM_2.5_: IRR of 1.51 (95% CI: 1.40 to 1.62) per 5 µg/m³ increase.
22	Yuchi et al. (2022) [51]	ADHD identified by hospital records, physician visits, and prescriptions using a well-defined criterion for ADHD cases, adjusted for sensitivity and specificity.	Incidence of ADHD was 3.3%. For PM_2.5_, an IQR increase in exposure was associated with a 11% increase in the risk of ADHD. Greenness showed a protective effect with a 10% decrease in ADHD risk per IQR increase in greenness exposure. NO2 and noise showed no significant associations with ADHD risk.	Greenness was associated with a lower incidence of ADHD (HR: 0.90 [0.81–0.99] per interquartile range increment of greenness), while PM_2.5_ was associated with an increased incidence (HR: 1.11 [1.06–1.17] per interquartile range increment). Male 1.14 [1.08-1.21] with 2.25 μg/m3 increase in PM_2.5_, Female 1.03 [0.94-1.13] with 2.11μg/m3 increase in PM _2.5_ NO_2_ and noise were not significantly associated. HR 1.01 [.96–1.07]
23	Li et al.(2023) [52]	ASD was measured by the Children’s Social Behavior Questionnaire and the Adult Social Behavior Questionnaire. ADHD was assessed using the Child Behavior Checklist and the Adult Behavior Checklist.	Higher levels of PM exposure were associated with more severe symptoms of ASD and ADHD, although these associations decreased over time. No consistent associations were observed for other pollutants or noise.	PM_2.5_: Increase of 5 µg/m³ associated with an increase in ADHD severity score of 0.22 points (95% CI: 0.05, 0.39). PM_10_: Increase of 10 µg/m³ associated with an increase in ASD severity score of 0.28 points (95% CI: 0.11, 0.45). O_3_, SO_2_, NO_2_: No significant associations.
24	Zhang et al. (2022) [34]	Assessed using the CBCL which compares child behavior patterns to age and gender norms.	Proximity to coal-fired power plants was associated with higher indoor levels of PM_10_ and increased neurobehavioral problems among children, particularly those living within 10 miles of two major power plants.	Distance to power plant: Significant negative regression coefficients with neurobehavioral symptoms such as affective problems, anxiety problems, ADHD, and social problems. PM_10_: Not directly associated with neurobehavioral outcomes in the regression models but played a role in exposure assessment. Log value of PM_10_ (μg/m3) was 0.631 on multiple regression model.
25	Zhou et al. (2023) [35]	ADHD symptoms (score ≥15), ADHD tendencies (score 11 ≤ score ≤14), and attention-deficit/hyperactivity problems (score ≥11).	Incidence of ADHD was 2.3% and ADHD-T 5.6%. Long-term ozone exposure was associated with an increased risk of ADHD and its subcategories among preschool and school-age children.	ADHD: Each IQR increase in ozone concentration associated with an OR of 1.13 (95% CI: 1.05–1.21), p=0.001. ADHD-T: Each IQR (2.47 μg/m3) increase in ozone concentration associated with an OR of 1.08 (95% CI: 1.03–1.14), p=0.001. ADHP: Each IQR increase in ozone concentration associated with an OR of 1.09 (95% CI: 1.05–1.14), p < 0.001. Non-breastfeed ADHD 1.22 (1.09, 1.36)

Association Between PM_2.5_ and ADHD

In Chen et al.'s cohort study using data from Spain, PM_2.5_ exposure during specific windows in mid-gestation and early toddlerhood was associated with increased hyperactivity symptoms at ages 5 and 7 [38]. The RR for PM_2.5_ exposure during the 1.2-2.9-year age range was 2.78 (95%CI: 2.02-3.82). However, for the age range of 3.8-4.1 years, the RR was 0.81 (95%CI: 0.74-0.89).

In Liu et al.'s large cross-sectional study from China, the association between PM_2.5_ exposure and ADHD was evaluated among school-aged children [44]. The OR for PM_2.5 _exposure was 1.65 (95%CI: 1.45-1.88) per 10 μg/m³ increase. Stratified analyses showed that both boys and girls were similarly affected, with ORs of 1.65 and 1.61 per 10 μg/m³ increase, respectively.

 Chang et al.'s birth cohort study in Taiwan showed that PM_2.5_ exposure from the prenatal and early postnatal periods significantly increased the risk of developing ADHD [29]. The HR for PM_2.5_ exposure during the third year of life was 1.87 per 10 μg/m³ increase (95%CI: 1.53-2.29). The dose-response relationship was evident, with higher levels of PM_2.5_ correlating with a substantially higher risk of ADHD, especially during the first three years of life.

A retrospective cohort study in Taiwan found that higher PM_2.5_ levels during early childhood were significantly associated with an increased risk of ADHD [40]. The adjusted HR (aHR) for PM_2.5_ in the highest quartile of exposure compared to the lowest was 1.79 (95% CI: 1.58-2.02), indicating a dose-dependent relationship where higher PM_2.5_ exposure correlated with a greater risk of developing ADHD. A population-based birth cohort study in Canada found that an interquartile range (IQR) increase in PM_2.5_ exposure was associated with an 11% increase in ADHD risk, with a HR of 1.11 (95%CI: 1.06-1.17) per IQR increment [51].

Rivas et al.'s study within the BREATHE (Building Resilient Environments for Air and Total HEalth) project in Spain assessed the long-term effects of PM_2.5_ exposure on cognitive outcomes [32]. The cumulative effect of a 10 µg/m³ increase in PM_2.5_ from birth to the seventh year of life resulted in a reduction of working memory scores by -19.50 points and poorer performance in attention tasks.

A longitudinal cohort study by Roberts et al. in the United Kingdom investigated the effects of air pollution on mental health outcomes, including ADHD [47]. While PM_2.5_ exposure at age 12 was associated with increased odds of depression at age 18, no significant association was found between PM_2.5_ exposure and ADHD outcomes. The OR for ADHD with PM_2.5_ exposure in the full model was 1.16 (95%CI: 0.64-2.10), indicating no significant relationship.

In the Danish cohort study by Thygesen et al., the incidence of ADHD was found to be significantly associated with early childhood exposure to PM_2.5_ [50]. An increase of 5 μg/m³ in PM_2.5_ levels was associated with an incident rate ratio (IRR) of 1.51 (95%CI: 1.40-1.62), showing a strong relationship between PM_2.5_ exposure and ADHD risk during childhood.

Sunyer et al.'s follow-up study in Spain assessed the effects of PM_2.5_ exposure on attention processes in school children [49]. Children exposed to higher daily levels of PM_2.5_ showed slower response times and reduced attention performance. Specifically, a 10 µg/m³ increase in PM_2.5_ resulted in a 1.1-month developmental retardation in response speed.

Fuertes et al.'s cohort study from Germany investigated the relationship between PM_2.5_ exposure and hyperactivity/inattention in children [31]. The OR for hyperactivity/inattention associated with PM_2.5_ exposure at 10 years of age was 1.12 (95%CI: 1.01-1.23), and at 15 years of age, it was 1.11 (95%CI: 1.01-1.22).

**Figure 2:**
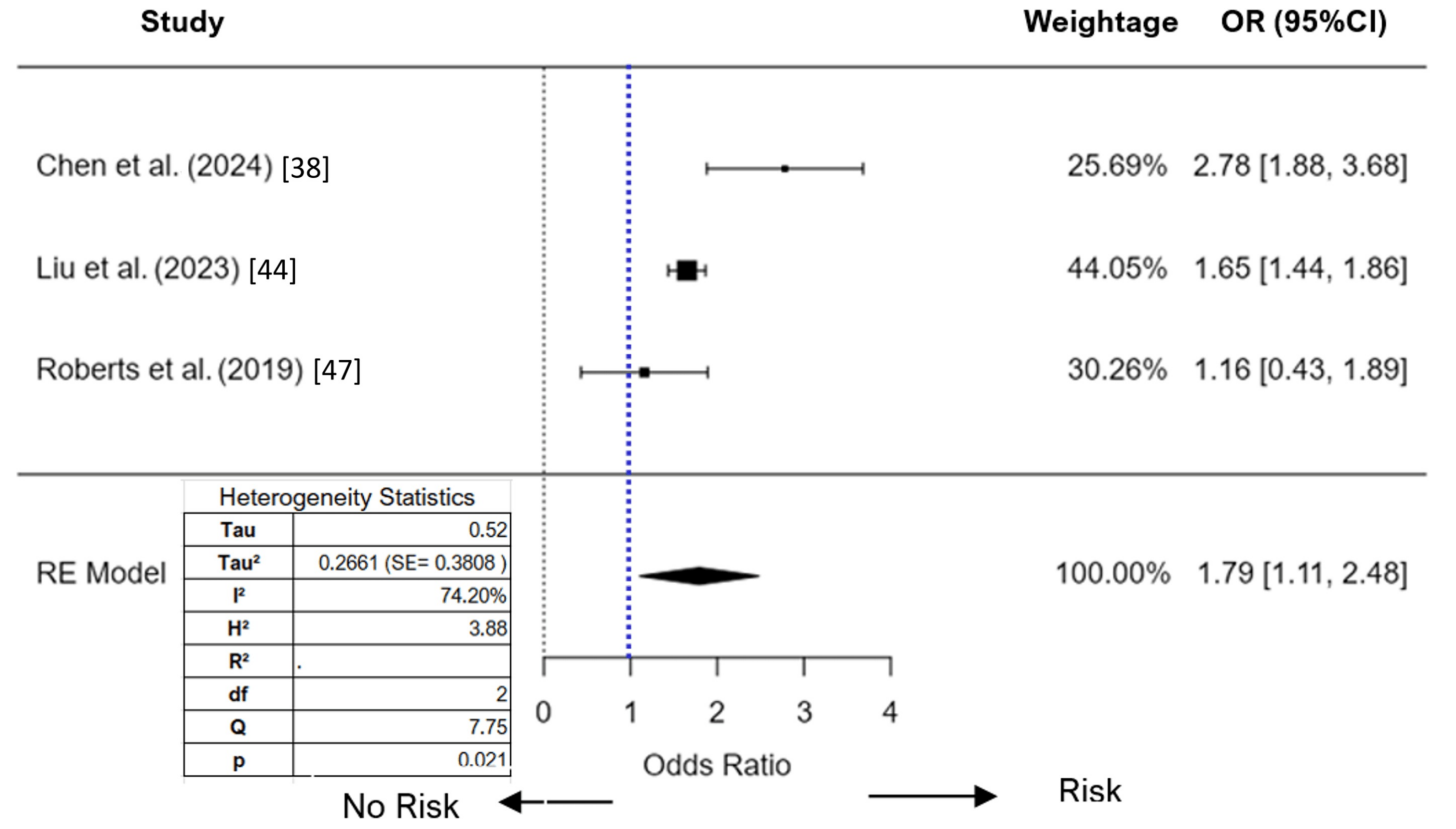
Forest plot (random effect) of the association between PM2.5 exposure and ADHD (odds ratios) RE: random effect; SE: standard error; ADHD: attention-deficit/hyperactivity disorder; PM_2.5_: particulate matter 2.5 microns or less References: [38], [44], [47]

**Figure 3:**
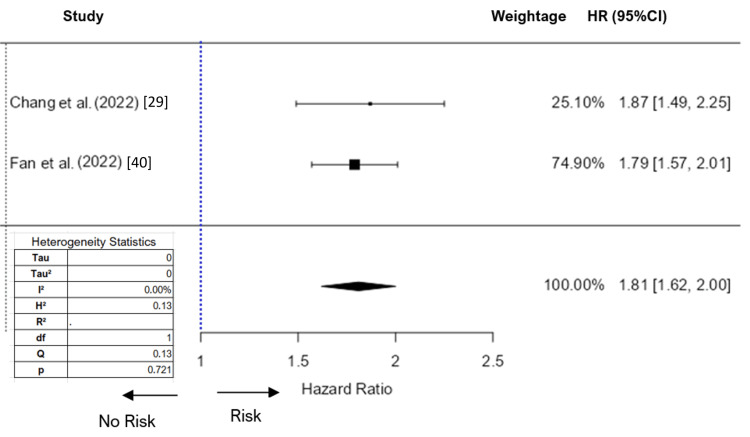
Forest plot (fixed effect) of the association between PM2.5 exposure and ADHD (hazard ratio) HR: hazard ratio; FE: fixed effect; SE: standard error References: [29], [40]

We conducted a meta-analysis to examine the association between PM_2.5_ exposure and adverse health outcomes, utilizing two different effect sizes (Figures 2, 3): HR and OR. Given the differences in the nature and interpretation of these effect sizes, separate analyses were performed to ensure the robustness of the findings and to capture the nuances in how PM_2.5_ exposure impacts ADHD over different study designs and follow-up periods. The random-effects model, based on three studies (k = 3), produced an overall estimated OR of 1.79, with a standard error of 0.350. The Z-value of 5.1198, coupled with a p-value of less than 0.001, indicates that the association between PM_2.5_ exposure and the studied outcome is statistically significant. The 95%CI for the OR ranges from 1.11 to 2.49, suggesting that there is a significant association between PM_2.5_ exposure and the occurrence of ADHD, with the true OR lying within this range.

The Tau² value and I² statistic was 0.27 and 74.2%, respectively. This level of heterogeneity is considered substantial, indicating that the effect sizes vary considerably among the studies. The Q statistic was 7.75 with a p-value of 0.021, further supporting the presence of significant heterogeneity. Since the number of studies was less than 10, we didn’t check for publication bias. In the meta-analysis of PM_2.5_ on ADHD using hazard ratios, we initially included three studies. The analysis revealed a high level of heterogeneity with an I² value of 95.8%. To address this high value, we conducted outlier and influential case diagnostics. These diagnostics identified the study by Yuchi et al. [51] as an influential outlier. After removing this study from the analysis, the I² value decreased to 0%. We decided to apply a fixed-effects model for the meta-analysis of the remaining two studies. The analysis produced an estimate of 1.81, with a standard error of 0.097, and a significant Z-value of 18.6 (p < 0.001). The 95%CI ranged from 1.61 to 2.0, indicating a strong and statistically significant association between PM_2.5_ exposure and the risk of ADHD.

Association Between PM_10_ and ADHD

Li et al.'s study found that higher levels of PM_10_ exposure were associated with increased severity of ADHD symptoms [52]. An increase of 10 µg/m³ in PM_10_ was linked to an increase in autism spectrum disorder (ASD) severity score by 0.28 points (95%CI: 0.11, 0.45). Although the focus was on ASD, the study also noted a relationship between PM_10_ and ADHD severity.

In a retrospective cohort study conducted by Fan et al. in Taiwan, the authors found that higher exposure to PM_10_ during early childhood was significantly associated with an increased risk of ADHD [40]. The aHR for PM_10_ exposure in the highest quartile was 1.53 (95% CI: 1.37-1.70). In the cross-sectional study by Shim et al. from South Korea, higher PM_10_ concentrations were linked to an increased risk of ADHD [37]. For each 10 µg/m³ increase in PM_10_, the OR was 1.44 (95%CI: 1.02-2.02). This association was stronger for females (OR = 2.17, 95%CI: 1.00-4.72).

Zhang et al.'scommunity-based study conducted in the United States found that proximity to coal-fired power plants was associated with higher indoor levels of PM_10_, which in turn contributed to increased neurobehavioral problems in children [34]. While PM_10_ was not directly associated with ADHD outcomes in their regression models, the exposure was significant in assessing neurobehavioral outcomes.

Although Loftus et al.'s study mainly focused on NO_2_ exposure, PM_10_ exposure was also evaluated [45]. However, no association was found between PM_10_ levels and external behavioural outcomes in children, with an OR for PM_10_ exposure of 0.87 (95%CI: 0.46-1.64).

Markevych et al.'s study from Germany found that increased PM_10_ exposure was associated with a higher risk of ADHD [43]. Specifically, an increase of 10 µg/m³ in PM_10_ raised the relative risk of ADHD by 1.97 (95%CI: 1.35-2.86). Conducted in South Korea, the study by Min and Min found a significant association between increased exposure to PM_10_ and the incidence of ADHD [30]. The HR for PM_10_ was 1.17 (95%CI: 1.14-1.20) per 1 µg/m³ increase in exposure. In the highest tertile, the HR was 3.73 (95%CI: 2.76-5.03), showing a clear exposure-response relationship between higher PM_10_ levels and an increased risk of ADHD.

Fuertes et al.'s cohort study from Germany found a positive association between PM_10_ exposure and hyperactivity/inattention symptoms in children [31]. The OR for PM_10_ at 10 years of age was 1.05 (95%CI: 0.95-1.17), while at 15 years, the OR was 1.02 (95%CI: 0.93-1.11). Although not statistically significant, the trend suggested a potential link between PM_10_ exposure and hyperactivity/inattention symptoms.

In a twin cohort study from Sweden, the authors found no significant association between PM_10_ exposure and ADHD [42]. The adjusted OR for PM_10_ exposure at the age of nine years was 1.33 (95% CI: 0.73-2.41).On the other hand,Siddique et al.'s cross-sectional study conducted in India found a significantly higher prevalence of ADHD among children exposed to high PM_10_ levels [36]. The OR for children exposed to PM_10_ levels of 120-139 µg/m³ was 1.8 (95%CI: 1.1-3.6), while exposure to PM_10_ levels above 200 µg/m³ had an OR of 2.7 (95%CI: 1.4-5.5).

**Figure 4:**
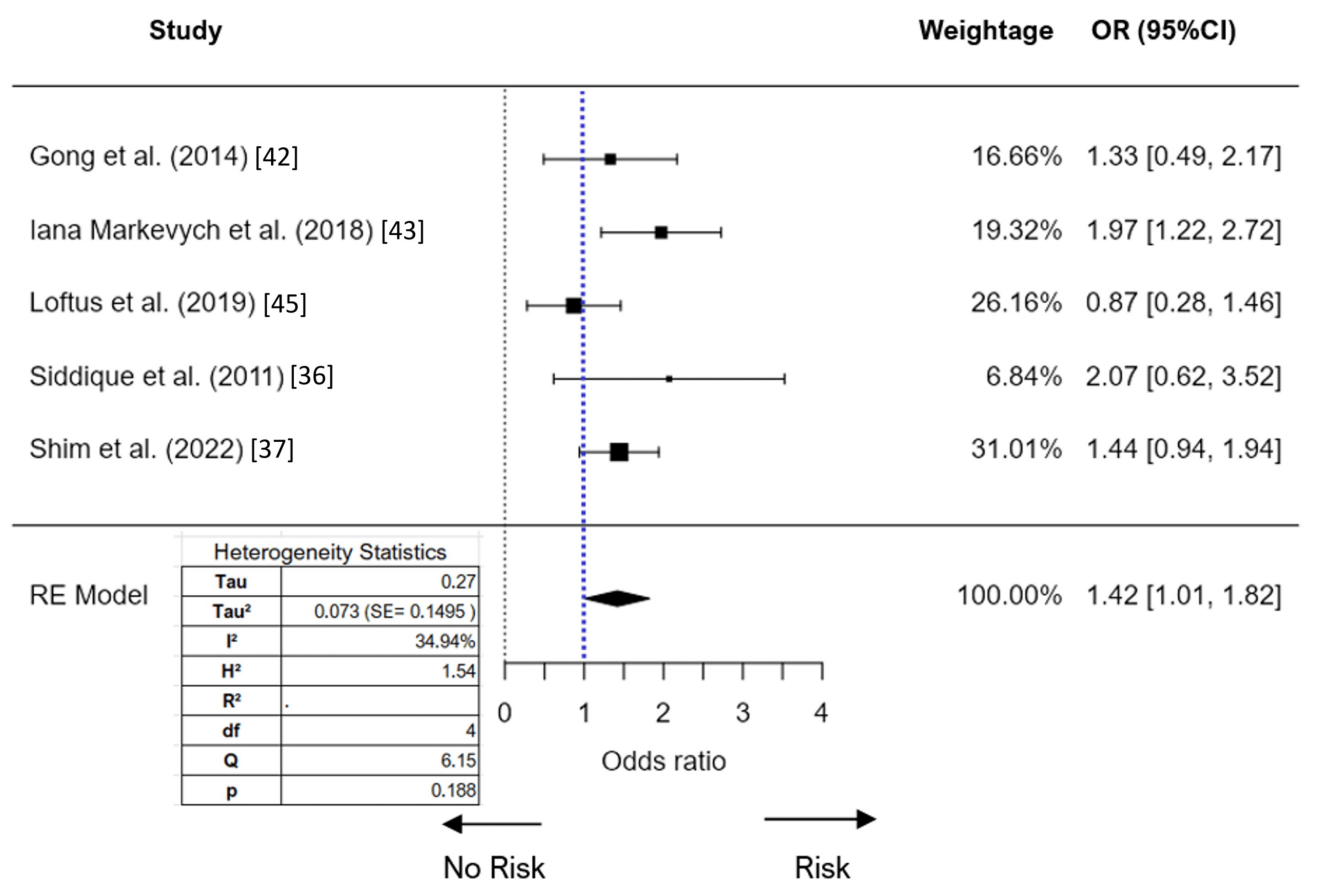
Forest plot (random effect) of the association between PM10 exposure and ADHD (odds ratio) RE: random effect; SE: standard error; ADHD: attention-deficit/hyperactivity disorder; PM_10_:  particulate matter 10 micrometers or less in diameter References: [42], [43], [45], [36], [37]

The meta-analysis of PM_10_ exposure and its association with ADHD initially included six studies (Figure 4), and a random-effects model was used which yielded an I² value of 76.6%. To address this issue, outlier and influential case diagnostics were conducted. The study by Min and Min [30] was identified as an influential outlier. Upon removing this study from the analysis, the I² value significantly decreased to 34.9%. The meta-analysis of PM_10_ exposure and ADHD produced an overall estimated OR of 1.42 (95%CI 1.01-1.82), indicating a significant association between PM_10_ exposure and the risk of ADHD, with a Z-value of 6.86 (p < 0.001). The publication bias assessment could not be studied due to the small number of studies.

Association Between NO_2_ and ADHD

In the longitudinal cohort study by Li et al. from the Netherlands (TRacking Adolescents' Individual Lives Survey (TRAILS)), the impact of higher levels of NO_2_ exposure on on ADHD symptom severity was assessed [52]. However, no significant association was found between NO_2_ and ADHD symptoms. Similarly, *i*n a population-based birth cohort study by Yuchi et al. from Canada, NO_2_ exposure was evaluated alongside other pollutants and found no significant association between NO_2_ exposure and ADHD incidence [51]. The HR for NO_2_ was 1.01 (95%CI: 0.96-1.07).

On the other hand, in Thygesen et al.'s large Danish prospective cohort study, early childhood exposure to NO_2 _was associated with an increased risk of developing ADHD [50]. The IRR for NO_2_ was 1.38 (95%CI: 1.35-1.42) per 10 µg/m³ increase. This significant association highlights the contribution of NO_2_ to the increased risk of ADHD, even after controlling for several potential confounders.

Roberts et al.'s longitudinal cohort study from the United Kingdom examined the relationship between NO_2_ exposure and ADHD at ages 12 and 18 [47]. No significant associations were found between NO_2_ exposure and ADHD outcomes. The OR for NO_2_ exposure and ADHD was 1.20 (95%CI: 0.69-2.09) in the full model.

In Sentis et al.'s population-based birth cohort study in Spain, prenatal and postnatal NO_2_ exposure was associated with impaired attentional function in children aged 4-5 years [48]. The study found an increase of 1.12 milliseconds in hit reaction time (HRT) standard error per 10 µg/m³ increase in prenatal NO_2_. Additionally, a 6% increase in omission errors was observed, though postnatal NO_2_ results showed only borderline significance.

In a longitudinal cohort study in the United States, NO_2_ exposure was associated with an increase in externalizing behaviours among children [45]. The study found that children from lower socioeconomic status (SES) backgrounds were particularly vulnerable to the effects of NO_2_. The OR for NO_2_ exposure and clinically significant externalizing behaviour was 1.96 (95%CI: 1.03-3.71), though no association was observed with ADHD specifically.

Markevych et al.'sstudy from Germany reported a significant association between NO_2_ exposure and ADHD incidence [43]. An increase of 10 μg/m³ in NO_2_ was associated with a RR of 1.32 (95%CI: 1.10-1.58). This association was found alongside increased risks with other pollutants.

In the prospective cohort study by Alemany et al. in Spain, NO_2_ exposure was associated with higher behavioural problem scores and reductions in cognitive function [28]. The study found a β value of 0.08 (95% CI: 0.03-0.13) per IQR increase in NO_2_ for behaviour problems. However, no significant association was found between NO_2_ and ADHD symptom scores, with an OR of 1.05 (95%CI: 0.95-1.16).

Forns et al.'s longitudinal cohort study in Spain investigated cognitive development in relation to NO_2_ exposure [41]. Persistent exposure to NO_2_ at school was negatively associated with cognitive development, particularly working memory. The coefficient for outdoor NO_2_ was -4.22 (95% CI: -6.22, -2.22) per IQR increase in NO_2_.In Forns et al.'s earlier cross-sectional study from Spain, in which NO_2_ exposure levels were measured at schools, the adjusted mean ratio (aMR) for outdoor NO_2_ exposure was 1.07 (95%CI: 1.01-1.14) for increased behavioural difficulties (Strengths and Difficulties Questionnaire (SDQ) score), though the association with ADHD symptoms (ADHD DSM-IV score) was not statistically significant, with an aMR of 1.03 (95% CI: 0.94-1.13) [33].

In a South Korean population-based cohort study, the HR for NO_2_ exposure and ADHD incidence was 1.03 (95% CI: 1.02-1.04) per 1 µg/m³ increase [30]. The study demonstrated a significant association between NO_2_ exposure and ADHD risk, with the highest tertile showing an HR of 2.16 (95% CI: 1.61-2.90) for NO_2_ exposure.On the other hand, in another twin cohort study from Sweden, which assessed the association of NO_2_ exposure with ADHD and ASD, no significant associations were found for NO_2_ exposure in relation to ADHD [42]. The aOR for NO_2_ exposure at nine years of age was 1.15 (95%CI: 0.62-2.15).

**Figure 5:**
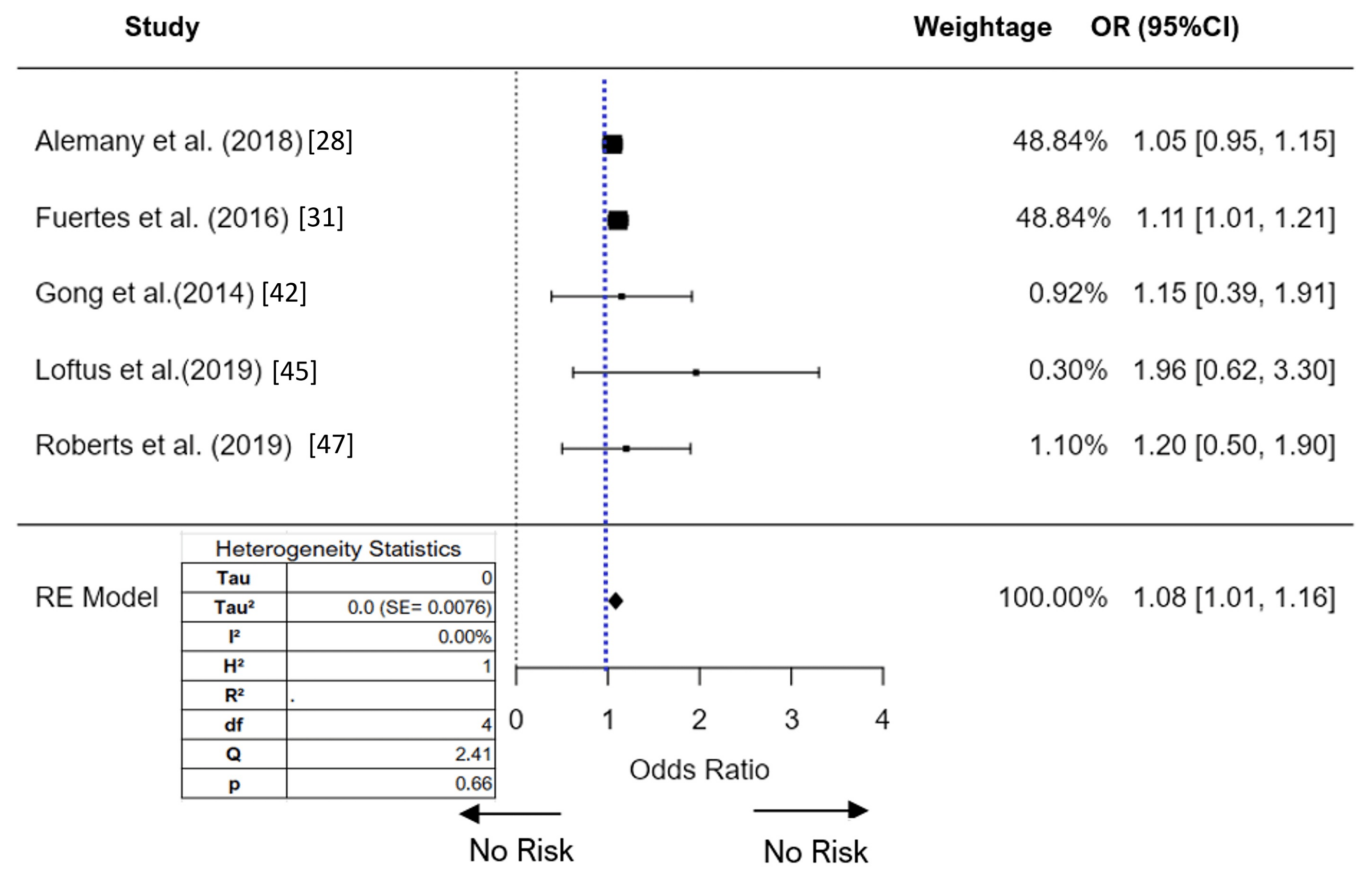
Forest plot (random effect) of the association between NO2 exposure and ADHD (odds ratios) RE: random effect; SE: standard error; NO_2_: nitrogen dioxide; ADHD: attention-deficit/hyperactivity disorder Reference: [28], [31], [42], [45], [47]

We performed a meta-analysis of NO_2_ exposure on ADHD using both HR and OR as effect sizes (Figure 5). Seven studies were included in the analysis, with five reporting ORs and two reporting HRs. Due to significant heterogeneity, particularly with an I^2^ value of 91.7% for the HR studies, the decision was made not to report the findings for HR, focusing instead on the OR results. The random-effects model, applied to the five studies reporting ORs, yielded an overall estimate of 1.08 (95%CI 1.01-1.16) with a standard error of 0.037. The Z-value of 28.97 and a p-value of less than 0.001 indicate a statistically significant association between NO_2_ exposure and ADHD. Heterogeneity statistics showed the I² value of 0% and a Q-statistic of 2.41 (p = 0.66).

Association Between SO_2_ and ADHD

In Li et al.'s longitudinal cohort study from the Netherlands (TRAILS), the impact of higher levels of SO_2_ exposure on ADHD and ASD symptom severity was assessed [52]. However, no significant associations were observed for SO_2_ in relation to ADHD or ASD symptoms. The study reported no meaningful increase in risk for SO_2_ exposure, with no significant relationship found between SO_2_ and ADHD outcomes. Similarly, in their sensitivity analyses and multi-pollutant models, SO_2_ exposure did not demonstrate significant effects on ADHD symptom severity.

On the other hand, in Siddique et al.'s cross-sectional study from India (Delhi), urban children exposed to higher levels of SO_2_ had a higher prevalence of ADHD compared to rural children [36]. However, the focus was primarily on PM_10_ exposure, and the specific OR for SO_2_ was not provided independently in this study. The general conclusion was that urban environments, with elevated levels of pollutants like SO_2_, PM_10_, and NO_2_, contributed to an increased risk of ADHD.

Association Between Ozone and ADHD

Li et al. conducted a longitudinal cohort study in the Netherlands involving 2,750 children (10-12 years old at the start, followed up to ages 24-28) which examined the effects of multiple pollutants, including ozone, on ADHD and ASD symptom severity [52]. However, the study found no significant associations between ozone exposure and either ADHD or ASD, suggesting that ozone did not play a notable role in exacerbating neurodevelopmental symptoms.

Zhou et al. conducted a cross-sectional study in China, part of the Seven Northeastern Cities (SNEC) study, which included 35,103 children aged 3-12 years [35]. This study found a significant association between long-term ozone exposure and the risk of ADHD. For every IQR increase in ozone concentration, the OR for ADHD increased to 1.13 (95%CI: 1.05-1.21, p = 0.001). Additionally, ADHD tendencies and attention-deficit/hyperactivity problems also showed a significant association with ozone exposure. For ADHD tendencies, each IQR increase of 2.47 µg/m³ in ozone was associated with an OR of 1.08 (95%CI: 1.03-1.14, p = 0.001), while for attention-deficit/hyperactivity problems, the OR was 1.09 (95%CI: 1.05-1.14, p < 0.001).

Association Between Black Carbon and ADHD

Alemany et al. conducted a prospective cohort study on 2,897 children aged 7-11 years in Spain, measuring the impact of polycyclic aromatic hydrocarbons (PAHs), black carbon, and NO_2_ on behaviour and cognitive development [28]. Black carbon levels were associated with smaller reductions in inattentiveness over time and higher behaviour problem scores, especially in children carrying the APOE e4 allele.

In a cross-sectional study of the same cohort (2,897 children aged 7-11 years) by Forns et al., elemental carbon and black carbon levels were measured in schools and positive associations was found with general behavioural problems and specific ADHD symptoms [33]. The adjusted mean ratio (aMR) for SDQ total difficulties scores for IQR increases in black carbon was 1.07 (95% CI: 1.03-1.12), indicating a higher incidence of behavioural difficulties linked to black carbon exposure. Similarly, black carbon exposure was associated with ADHD symptoms, with an aMR of 0.99 (95%CI: 0.93-1.07) for ADHD-DSM-IV scores.

Forns et al., in a longitudinal cohort study of 2,897 children, examined cognitive development, particularly working memory and inattentiveness, over a 3.5-year follow-up period [41]. The study found that persistent exposure to elemental carbon, as well as NO_2 _and PM_2.5_, was associated with negative impacts on working memory. Specifically, the coefficient for outdoor elemental carbon exposure on working memory development was -4.12 (95%CI: -5.68, -1.83)

In a prospective birth cohort study involving 174 children aged 7-14 years in the United States, Chiu et al. found that higher lifetime exposure to black carbon was associated with increased commission errors and slower HRT in the Conner’s Continuous Performance Test (CPT) [39]. The beta values for commission errors with increased black carbon exposure were 8.88 (95%CI: 2.64, 15.1) in the second quartile and 9.17 (95%CI: 1.54, 16.8) in the third quartile, compared to the first quartile. For HRT, beta values for the second and third quartiles were 10.1 (95%CI: 0.42, 19.8) and 7.9 (95%CI: -6.24, 16.8), respectively.

Newman et al.,in their prospective birth cohort study of 762 children in the United States, found that early-life exposure to higher levels of elemental carbon attributable to traffic (ECAT) was significantly associated with an increased risk of hyperactivity at age 7 [46]. Children in the highest tertile of ECAT exposure had an aOR of 1.7 (95%CI: 1.0-2.7) for hyperactivity compared to those with lower exposure.

For a forest plot to be meaningful in a meta-analysis, at least two studies with an outcome measure (which was taken into consideration), such as an OR or HR, need to be included as a prerequisite. Because of this requirement, a meta-analysis was not performed for SO_2_, ozone, and black carbon, as there were insufficient studies reporting these outcome measures for these pollutants.

Discussion

General Interpretation of the Results in the Context of Other Evidence

The results from this systematic review reinforce the growing body of evidence linking PM_2.5_ exposure to an increased risk of ADHD and other neurodevelopmental disorders in children. Across multiple studies, there is a consistent and significant association between PM_2.5_ exposure and ADHD, supporting the hypothesis that early-life exposure to fine particulate matter can have lasting effects on cognitive and behavioural development.

For instance, Chen et al. observed that specific windows of PM_2.5_ exposure during mid-gestation and early toddlerhood were linked to a significant increase in hyperactivity symptoms in children at ages 5 and 7 [38]. Similarly, Liu et al. found a significant increase in ADHD risk among school-aged children in China, suggesting that both boys and girls are affected equally by PM_2.5_ exposure [44]. Studies by Chang et al. [29] and Fan et al. [40], both conducted in Taiwan, also support these findings. They observed a dose-response relationship between high PM_2.5_ exposure levels and greater risk of developing ADHD. Similarly, Yuchi et al., in a population-based birth cohort study in Canada, found a significant increase in ADHD risk with increasing PM_2.5_ exposure [51]. However, Roberts et al. from the United Kingdom reported no significant association between PM_2.5_ exposure and ADHD outcomes, highlighting the variability in the relationship between PM_2.5_ exposure and ADHD risk across different populations and age groups [47]. This discrepancy could be attributed to differences in study designs, population characteristics, and exposure assessment methods. Rivas et al. found that cumulative exposure to PM_2.5_ from birth to the seventh year of life resulted in a significant reduction in working memory scores and poorer performance in attention tasks [32]. Sunyer et al. also reported that school children exposed to higher daily levels of PM_2.5_ exhibited slower response times and reduced attention performance, with the cognitive delay manifesting as slower developmental progress in response speed [49]. These findings suggest that the effects of PM_2.5_ exposure extend beyond ADHD to affect broader cognitive functions such as memory and attention processing. The association between PM_2.5_ exposure and hyperactivity/inattention were further supported by Fuertes et al., who found a consistent trend of increased hyperactivity and inattention symptoms in children exposed to higher PM_2.5_ levels [31]. Although the ORs in their study did not reach statistical significance, the findings suggest a potential link between long-term PM_2.5_ exposure and attention-related behaviours that may persist through adolescence.

The results of the meta-analysis on PM_2.5_ exposure and ADHD underscore a significant association between exposure to fine particulate matter and the increased risk of ADHD in children. This finding aligns with several studies highlighting the neurodevelopmental risks posed by PM_2.5_ exposure. For instance, the observed link between PM_2.5_ and ADHD is consistent with research by Rosi et al., which reported that air pollution exposure, including PM_2.5_, contributes to cognitive deficits and developmental disorders [53]. Similarly, Zhang et al. found that children exposed to higher levels of PM_2.5_ showed worse neurobehavioral outcomes, reinforcing the broader body of evidence suggesting detrimental effects of PM_2.5_ on neurodevelopment [34]. In contrast, while Huang et al. also found associations between air pollution and cognitive deficits, their study highlighted a more complex interaction where certain confounding factors, such as SES and concurrent exposure to other pollutants, could moderate the strength of the association [54]. This suggests that while PM_2.5_ is a significant risk factor, other environmental or individual-level factors may contribute to the variability seen in different studies. Despite the strong association found in this meta-analysis, the high heterogeneity among the included studies reflects the complexity of measuring the effects of PM_2.5_. Factors such as differences in exposure levels, diagnostic criteria for ADHD, and geographical variability could explain the inconsistency in effect sizes. Additionally, unlike the current analysis, some studies, such as those referenced by Rosi et al. [53], emphasize the broader cognitive impacts beyond ADHD, suggesting that PM_2.5_ might affect various neurodevelopmental outcomes depending on exposure timing and intensity.

The findings from this systematic review reinforce the association between PM_10 _exposure and the increased risk of ADHD and other neurodevelopmental issues in children. The significant association between PM_10_ exposure and ADHD is supported by several studies. Li et al. found that higher PM_10_ levels were linked to increased severity of ADHD symptoms, indicating a strong relationship between particulate matter and neurodevelopmental outcomes [52]. Fan et al. further reinforced this link by showing a significant increase in ADHD risk with higher childhood exposure to PM_10_ in Taiwan [40]. Shim et al. also demonstrated a significant association between PM_10_ and ADHD risk in South Korea, with the effect being more pronounced in females, suggesting a potential gender difference in neurodevelopmental vulnerability [37]. Similarly, Markevych et al. [43] and Min and Min [30] from Germany and South Korea, respectively, confirmed that higher PM_10_ exposure was significantly associated with an increased risk of ADHD, with a clear dose-response relationship in the latter study. Siddique et al. also found that children in India exposed to higher PM_10_ levels had a significantly increased prevalence of ADHD, particularly in areas with the highest pollution levels [36].

In contrast, studies by Loftus et al. [45] and Gong et al. [42] found no significant association between PM_10_ exposure and ADHD. Loftus et al., while focused on NO_2_, did not find any significant relationship between PM_10_ and external behaviour, including ADHD, in their cohort from the United States [45]. Gong et al., in a Swedish twin cohort, similarly found no notable effect of PM_10_ on ADHD risk, suggesting that the relationship between PM_10_ and neurodevelopment might vary based on environmental or genetic factors [42]. Meanwhile, Fuertes et al., in Germany, showed a potential trend of increased ADHD risk with higher PM_10_ exposure, although their findings were not statistically significant [31]. This highlights the possibility of an association that could become clearer with longer follow-up periods or larger sample sizes, further emphasizing the need for continued research into the effects of PM_10_ on neurodevelopment.

The meta-analysis of PM_10_ exposure and ADHD found a significant association, consistent with prior research. Huang et al. similarly reported that air pollution, including PM_10_, negatively impacts neurodevelopment, reinforcing the meta-analysis findings [54]. Rosi et al. also linked particulate matter to behavioural disorders, including ADHD, supporting the broader evidence of air pollution’s role in neurodevelopmental issues [53]. In contrast, Zhang et al. found less direct evidence of PM_10_'s effect on ADHD but highlighted cognitive impairments related to pollution exposure [34].

Several studies reported significant associations between NO_2_ exposure and ADHD risk. Thygesen et al. found that higher childhood exposure to NO_2_ was linked to a significantly increased risk of ADHD in a Danish cohort, supporting the idea that early-life exposure to traffic-related air pollutants can impact neurodevelopment [50]. Min and Min also observed a dose-response relationship between NO_2_ exposure and ADHD incidence, particularly in populations exposed to higher levels of air pollution [30]. Similarly, Markevych et al. [43] from Germany and Loftus et al. [45] from the United States found significant associations between NO_2_ exposure and increased risk of ADHD or broader behavioural issues, with the latter study highlighting the vulnerability of children from lower SES backgrounds. Alemany et al. further supported the link between NO_2_ exposure and behavioural problems, although they did not find a significant association with ADHD specifically [28]. Some studies presented mixed or non-significant findings. Forns et al. showed that while NO_2_ exposure was associated with increased behavioural difficulties and impaired cognitive function, the direct link to ADHD symptoms was not statistically significant [33,41]. Roberts et al. found no significant associations between NO_2_ exposure and ADHD outcomes at ages 12 and 18, suggesting variability in NO_2_’s impact based on age and exposure methods [47]. Conversely, studies by Li et al. [52], Yuchi et al. [51] and Gong et al. [42] found no significant association between NO_2_ exposure and ADHD outcomes, highlighting the inconsistency in findings across different populations and environmental contexts. These conflicting results suggest that the impact of NO_2_ on ADHD may vary based on several factors, including population characteristics and exposure timing.

The meta-analysis on NO_2 _exposure and ADHD demonstrated a significant association, with no notable heterogeneity across studies, indicating consistent findings. This aligns with Huang et al., who highlighted the adverse effects of NO_2_ on neurodevelopment, including attention-related issues [54]. Rosi et al. also supported the role of NO_2_ in contributing to behavioural disorders, underscoring the pollutant’s potential to increase ADHD risk [53]. However, Zhang et al. emphasized the broader cognitive impacts of air pollution, without a direct focus on NO_2_’s specific role in ADHD development [34].

Siddique et al. found that urban children in Delhi exposed to higher levels of SO_2_ had a greater prevalence of ADHD compared to rural children, although SO_2_'s specific impact was not isolated from other pollutants like PM_10_ and NO_2 _[36]. In contrast, Li et al., in a longitudinal cohort study from the Netherlands, found no significant association between SO_2_ exposure and ADHD or ASD symptoms [52]. Their findings, consistent across sensitivity analyses, suggest SO_2 _may not be a significant contributor to ADHD risk.

In the study by Zhou et al. from China, long-term ozone exposure was significantly associated with an increased risk of ADHD [35]. Each IQR increase in ozone concentration was linked to higher odds of ADHD and related behavioural problems, highlighting a dose-response relationship. In contrast, Li et al., in a longitudinal cohort study from the Netherlands, found no significant association between ozone exposure and ADHD or ASD symptoms, suggesting that the role of ozone in neurodevelopmental disorders may vary across different populations and environmental contexts [52].

Black carbon exposure has been consistently linked to negative neurodevelopmental and behavioural outcomes in children. Alemany et al. reported increased behavioural problems and slower improvements in inattentiveness among children exposed to higher black carbon levels [28]. Forns et al. found that black carbon exposure was associated with elevated behavioural difficulties and ADHD symptoms [41]. Similarly, in their earlier study, Forns et al. had observed that long-term exposure to black carbon impaired cognitive development, particularly working memory [33]. Chiu et al. [39] noted that higher lifetime black carbon exposure was linked to increased commission errors and slower reaction times in children, while Newman et al. [46] identified a significant association between early-life black carbon exposure and a heightened risk of hyperactivity by age 7.

Limitations of the Evidence Included in the Review

The evidence included in this review is subject to several limitations that affect its completeness, applicability, and certainty. One of the key limitations is the inconsistency in the measurement of both exposure and outcomes across the included studies. While some studies used validated methods like land-use regression models or direct monitoring for pollutant exposure, others relied on less precise measures, such as proximity to pollution sources or indirect estimations, leading to potential inaccuracies. Additionally, ADHD diagnoses were not consistently applied; some studies used clinical assessments based on DSM criteria, while others relied on parent or teacher-reported questionnaires, introducing variability in outcome measurements.

Many of the studies included in this review were cross-sectional in nature, limiting the ability to draw causal inferences regarding the long-term impact of air pollution exposure on ADHD. Longitudinal data were sparse, and the available studies that did follow participants over time often lacked adequate follow-up periods to assess the full spectrum of neurodevelopmental effects. This shortfall impacts the ability to fully understand the temporal relationship between exposure to pollutants and the development of ADHD.

The populations studied also present limitations in terms of generalizability. The majority of studies focused on children from urban areas, and many lacked representations from diverse socioeconomic or geographical backgrounds, potentially limiting the applicability of the findings to broader populations. Selection bias may also have influenced the results, as many studies did not adequately address potential confounding factors like co-exposure to other pollutants or environmental stressors that could independently influence ADHD risk.

Furthermore, several studies had small sample sizes, which may lead to imprecise effect estimates and limit the statistical power of the findings. Some studies combined outcomes for multiple pollutants, making it difficult to isolate the specific effects of individual pollutants like PM_2.5_, PM_10_, NO_2_, or SO_2_.

Finally, there is the possibility of publication bias, as studies that report significant associations between pollutants and ADHD may be more likely to be published, while studies with null results are less likely to be disseminated. Despite statistical methods used to detect publication bias, this remains a concern that could influence the overall conclusions of the review. The relatively small number of studies included in the meta-analyses for each pollutant (PM_2.5_, PM_10_, NO_2_) may reduce the precision of the pooled estimates. Furthermore, the heterogeneity observed in some pollutant analyses, such as PM_10_, underscores the variability in study designs, population characteristics, and exposure measurement methods, which could influence the results. The exclusion of non-English studies and the reliance on observational data further limited the generalizability of the findings.

Limitations of the Review Processes Used

While this SRMA adhered to a robust protocol and PRISMA guidelines, there are some limitations in the review processes that could affect the comprehensiveness and validity of the findings. The review included only studies published in English, which may have led to the exclusion of relevant studies published in other languages. This could have particularly affected the inclusion of research from non-English-speaking countries, where air pollution exposure and its health impacts may differ due to varying environmental and socio-economic factors. Although we conducted a comprehensive search across several major databases (PubMed, Web of Science, Embase, OvidSP), it is possible that we missed some relevant studies. The search strategy, while extensive, did not include grey literature sources such as unpublished studies, reports, or theses. This may have introduced publication bias, as studies with non-significant results are less likely to be published in peer-reviewed journals.

The risk of bias was assessed using the JBI critical appraisal tools tailored for different study designs. The JBI cutoff scores were determined through a thorough discussion among the reviewers. Although this approach minimizes bias, the subjective nature of some assessments, particularly around confounding and exposure measurement, may still introduce variability in the evaluation. Despite efforts to contact study authors for full-text access, some articles could not be retrieved, and their exclusion may have led to the omission of relevant data. These missing studies could potentially impact the overall results, particularly in terms of providing a more comprehensive assessment of the evidence. However, both the title and abstract screening were conducted by two independent reviewers, ensuring that no relevant studies were inadvertently excluded. This dual-screening process significantly reduces the likelihood of missing eligible studies, a key strength in minimizing selection bias.

Additionally, limitations in the original observational studies, such as residual confounding, may have influenced the meta-analysis. Finally, we could not perform certain subgroup analyses due to data variability, which could affect the depth of the review's conclusions.

Implications of the Results for Practice, Policy, and Future Research

The strong association between ambient air pollution, particularly PM_2.5_, PM_10_, and NO_2_, and ADHD risk in children highlights the need for healthcare professionals to consider environmental exposures when assessing and managing ADHD in paediatric populations. Clinicians should be aware of the potential role that air pollution plays in exacerbating neurodevelopmental disorders and counsel families, especially those in high-pollution areas, on strategies to minimize exposure. This may include recommendations for indoor air purification, limiting outdoor activities during peak pollution periods, and advocating for cleaner environments at home and school.

The findings emphasize the urgent need for stricter air quality regulations, especially in urban areas with high levels of particulate matter and traffic-related pollutants. Policymakers should prioritize reducing ambient air pollution through legislation aimed at lowering emission levels from industrial sources, traffic, and other contributors. Given the clear impact of air pollution on neurodevelopment, policies targeting early-life exposure are particularly crucial. Establishing better air quality monitoring systems in schools and residential areas will also be key to protecting vulnerable populations, such as children, from the detrimental effects of pollution.

Despite strong evidence linking air pollution to ADHD, gaps remain. Future studies should focus on longitudinal designs that can track the long-term neurodevelopmental impacts of pollution exposure over time. Additionally, more research is needed to explore the mechanisms underlying the relationship between specific pollutants and ADHD, especially in diverse populations and environments. Investigating the role of genetic predispositions, socio-economic factors, and gender differences will help refine risk assessments and intervention strategies. Further research is also needed on the efficacy of interventions aimed at reducing pollution exposure and mitigating its neurodevelopmental impacts. Expanding research to regions with limited data and including pollutants like ozone and sulphur dioxide will provide a more comprehensive understanding of the global burden of air pollution on child health.

## Conclusions

This SRMA demonstrates a significant association between ambient air pollution, particularly PM_2.5_, PM_10_, and NO₂, and an increased risk of ADHD in children. The findings underscore the need for targeted public health interventions to reduce air pollution exposure, particularly in urban and high-pollution areas where children are most vulnerable. Policymakers should implement stricter air quality regulations and promote preventive measures to protect children from neurodevelopmental harm. Further research is needed to explore the long-term effects of air pollution on ADHD and to better understand the mechanisms driving this association.

## Appendices

Availability of data, code, and other materials

The data used for the analyses in this review, including extracted data from included studies and materials used, are publicly available on the Open Science Framework (OSF) at the following link: https://osf.io/7vq5p/?view_only=c8341722e81240b3b56fa68b9579a109
